# Proteomics Analysis of Proteotoxic Stress Response in In-Vitro Human Neuronal Models

**DOI:** 10.3390/ijms25126787

**Published:** 2024-06-20

**Authors:** Ayodele Alaiya, Bothina Mohammed Alharbi, Zakia Shinwari, Mamoon Rashid, Tahani H. Albinhassan, Abderrezak Bouchama, Mai B. Alwesmi, Sameer Mohammad, Shuja Shafi Malik

**Affiliations:** 1 Cell Therapy & Immunobiology Department, King Faisal Specialist Hospital and Research Centre, Riyadh 11211, Saudi Arabia; 2 Experimental Medicine Department, King Abdullah International Medical Research Center, King Saud bin Abdulaziz University for Health Sciences, Ministry of National Guard Health Affairs, Riyadh 11426, Saudi Arabia; 3 Department of AI and Bioinformatics, King Abdullah International Medical Research Center, King Saud bin Abdulaziz University for Health Sciences, MNGHA, Riyadh 11426, Saudi Arabia; 4 Zoology Department, College of Science, King Saud University, Riyadh 12372, Saudi Arabia; 5 Medical-Surgical Nursing Department, College of Nursing, Princess Nourah bint Abdulrahman University, Riyadh 11671, Saudi Arabia

**Keywords:** neurons, proteostasis, heat shock, proteotoxic stress, stress response, proteomics

## Abstract

Heat stroke, a hazardous hyperthermia-related illness, is characterized by CNS injury, particularly long-lasting brain damage. A root cause for hyperthermic neurological damage is heat-induced proteotoxic stress through protein aggregation, a known causative agent of neurological disorders. Stress magnitude and enduring persistence are highly correlated with hyperthermia-associated neurological damage. We used an untargeted proteomic approach using liquid chromatography–tandem mass spectrometry (LC-MS/MS) to identify and characterize time-series proteome-wide changes in dose-responsive proteotoxic stress models in medulloblastoma [Daoy], neuroblastoma [SH-SY5Y], and differentiated SH-SY5Y neuron-like cells [SH(D)]. An integrated analysis of condition–time datasets identified global proteome-wide differentially expressed proteins (DEPs) as part of the heat-induced proteotoxic stress response. The condition-specific analysis detected higher DEPs and upregulated proteins in extreme heat stress with a relatively conservative and tight regulation in differentiated SH-SY5Y neuron-like cells. Functional network analysis using ingenuity pathway analysis (IPA) identified common intercellular pathways associated with the biological processes of protein, RNA, and amino acid metabolism and cellular response to stress and membrane trafficking. The condition-wise temporal pathway analysis in the differentiated neuron-like cells detects a significant pathway, functional, and disease association of DEPs with processes like protein folding and protein synthesis, Nervous System Development and Function, and Neurological Disease. An elaborate dose-dependent stress-specific and neuroprotective cellular signaling cascade is also significantly activated. Thus, our study provides a comprehensive map of the heat-induced proteotoxic stress response associating proteome-wide changes with altered biological processes. This helps to expand our understanding of the molecular basis of the heat-induced proteotoxic stress response with potential translational connotations.

## 1. Introduction

Among the detrimental determinants of climate change are the rising global temperature and the resultant heat waves [1]. Extreme environmental heat exposure through severe and enduring heat waves is a natural stressor impacting cellular physiology and eliciting pathophysiological responses [2,3]. Heat stroke is a life-threatening heat-related illness characterized by severe hyperthermia (core body temperature ≥ 40.5 °C) [4,5]. Progression to multiple organ dysfunction/injury syndrome (MODS), despite exhaustive treatment procedures, is observed in heat stroke patients [4,6]. An expected overall mortality rate exceeding 60% has been predicted even after optimal cooling and supportive treatment in intensive care [7]. Precise statistics on heat stroke-related morbidity and mortality are hampered by a lack of a standard definition for heat stroke and because of the underdiagnosis associated with this illness [8]. Some epidemiological studies in the United States reported 17.6–25.6 incidents of heat stroke per 100,000 population [9,10] and around 3300 deaths from 2006 to 2010 [11]. Taking into account the temperature severity and age of the patient, the mortality rate for heat stroke has been reported to vary between 10 and 70% with an overall global case fatality rate of about 12% [8,12]. Hyperthermia-induced cytotoxicity, compromised HSP expression, enhanced inflammation, and coagulation activation are the major contributors to heat injury pathogenesis, associated tissue damage, and death [4].

Heat stroke also leads to central nervous system (CNS) malfunctions like a coma, convulsions, and delirium, which can potentially lead to long-lasting and persistent neurological damage [13]. In fact, heat stroke encephalopathy is an early-stage, familiar, and primary heat stroke manifestation preceding the functional impediment of other organ systems [14]. Despite the survival and recovery from heat stroke, this heat stroke encephalopathy correlates with long-term motor and cognitive disabilities [15,16]. At the molecular level, proteotoxic stress and protein aggregation induce neurotoxicity and are causative agents of neurological disorders [17]. A direct correlation exists between protein misfolding, aggregation, and hyperthermia because of their evolutionary stabilization to function in limited and optimal growth temperature ranges [18]. A moderate temperature rise can induce protein unfolding and aggregation that can act as neurotoxic agents [18]. At the clinical level, initially, no specific neurological symptoms are detected in heat stroke patients and are accompanied by a standard brain image [14,19,20]. On the contrary, time-delayed brain images of heat stroke patients with lasting central nervous system dysfunction reveal cerebellar, hippocampal, and cortical damage [14]. Although aggregate accumulation is a common element in the severity and chronic behavior of neurological disorders, a higher rate of aggregate accumulation characterizes hyperthermia-induced aggregation [21]. These and other factors contribute to the persistence of hyperthermia-induced neurological defects [21], and the magnitude of hyperthermia-induced neuronal damage is proportionate with the duration and magnitude of the heat stress [13,21]. These considerations highlight the need to study heat-induced neuronal damage directly in heat-induced proteotoxic stress to help unravel the hyperthermia-induced underlying molecular mechanisms. Also, the delayed detection of brain damage emphasizes the need to identify biomarkers for early brain injury detection [13,14,17,18,19,20,21,22]. Proteomic techniques and bioinformatics analysis are potent tools for deciphering the molecular mechanisms underlying biological processes. This usually involves elucidating cellular proteome composition and establishing functional relationships between significantly expressing proteins. In the present study, we apply a label-free quantitative proteomics-based liquid chromatography–tandem mass spectrometry (LC-MS/MS) approach for a proteome-level analysis of the stress response in in vitro neuronal heat-induced proteotoxic stress models. Our group’s earlier stress response study based on time-series chaperone marker expression, protein aggregation measurement, and cell proliferation and viability established extreme [EX] and mild [MI] proteotoxic stress models [23]. We used three cell types to analyze the response to heat-induced proteotoxic stress over encompassing stress conditions: medulloblastoma [Daoy], neuroblastoma [SH-SY5Y], and differentiated SH-SY5Y neuron-like cells [SH(D)]. Medulloblastoma cells are cerebellar tumorous undifferentiated progenitor cells, while neuroblastoma cells are undeveloped nerve cells differentiated synthetically to terminal neurons. Therefore, the six groups analyzed include Daoy-EX, Daoy-MI, SH-SY5Y-EX, SH-SY5Y-MI, SH(D)-EX, and SH(D)-MI. Our previous study identified the 6–12 h post-recovery at 37 °C as critical to the expression of chaperone and co-chaperone proteins [24]. We, therefore, targeted two time points in the post-stress recovery phase: (i) 0H, immediately after the completion of stress exposure, and (ii) 6H, when chaperone expression peaks significantly—coupling the two time points to the six conditions above established 12 stress response states for analysis. This ensures the analysis of the dose-dependent stress response, critical to understanding hyperthermia-induced neurological damage’s dose-dependent persistence and severity. The untargeted proteomics approach will identify molecular fingerprints based on characterizing stress-induced proteome-wide modifications. The analysis of the association of these fingerprints with different molecular and functional pathways will develop an understanding of the complex interacting physiological events underlying the neuronal pathophysiology during hyperthermia-induced proteotoxic stress. 

## 2. Results

### 2.1. The Overall Proteomic Analysis

#### 2.1.1. Total and Differentially Expressed Proteins (DEPs)

**Figure 1:**
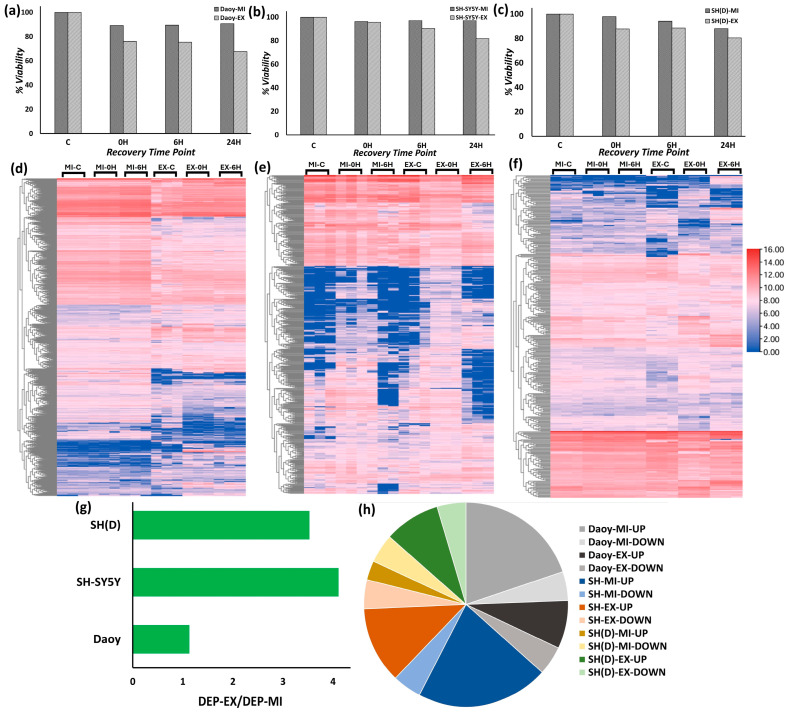
Model validation and overall proteomics analysis. Cell viability measurements using Trypan blue identify the differential stress levels in mild and extreme heat stress models in (**a**) Daoy, (**b**) SH-SY5Y, and (**c**) differentiated SH-SY5Y neuron-like cells. Both inter-condition and intercellular differences are observed. The results presented are an average of three experiments. Hierarchical clustering analysis helps visualize both condition-specific and studied time point-specific variations in (**d**) Daoy, (**e**) SH-SY5Y, and (**f**) differentiated SH-SY5Y neuron-like cells. (**g**) Compared to mild, the high number of DEPs in response to extreme heat stress. In Daoy cells, this change is less as the ratio is slightly more than 1, while in SH-SY5Y and differentiated SH-SY5Y neuron-like cells, it is around 3–4. (**h**) A pie chart representing cell and stress conditions specific up- and downregulated DEPs. Mostly, more upregulated proteins are identifiable in extreme heat stress response.

A total of 1903 proteins were detected both in Daoy-EX and Daoy-MI, 1749 and 1742, respectively, in SH-SY5Y-EX and SH-SY5Y-MI, and 1354 and 1357, respectively, in SH(D)-EX and SH(D)-MI. The differential nature of the stress in these models was validated through cell viability measurements (Figure 1a–c). Proteins identified with at least one unique peptide were used in the analysis. Cumulatively, 2957 proteins were detected in 18 independent biological samples. Of these 2957 proteins, 695 are common to the three cell types (Supplementary Figure S1a). Analysis by applying the ANOVA test and criteria of statistically significant difference in abundance (*p*-value ≤ 0.05) and a fold change of ≥2.0 identified the DEPs representing the proteome-wide global stress response changes. The analysis included six datasets per cell type and detected 1010 DEPs in Daoy cells, 623 in SH-SY5Y cells, and 365 in differentiated SH-SY5Y neuron-like cells. Thirty-two significant DEPs were represented across the three groups (Supplementary Figure S1b, Table 1). We utilized hierarchical clustering to compare the differential protein expression profiles visually. Clearcut proteome-wide differences are visible between extreme and mild heat stress conditions in the three cell types with intercell variation (Figure 1d–f). 

#### 2.1.2. Stress Condition-Specific Protein Expression Directionality

The next step involved identifying the regulation directionality for each cell type within each stress condition. A total of 282 DEPs in Daoy-MI (229 up and 53 down), 160 proteins in SH-SY5Y-MI (137 up and 23 down), and 80 proteins in SH(D)-MI (38 up and 42 down) show directionality. A similar analysis of the extreme stress revealed directionality in 320 proteins in Daoy-EX (200 up and 120 down), 687 proteins in SH-SY5Y-EX (499 up and 188 down), and 282 proteins in SH(D)-EX (186 up and 96 down). Overall, more proteins are differentially expressed (Figure 1g) and more proteins are upregulated (Figure 1h) during response to extreme heat stress. A list of all the DEPs, along with the mass spectrometry data, mean abundances, and results of the ANOVA, is accessible in Supplementary Table S1.

### 2.2. Stress Dose-Specific Overlapping and Unique Stress Response Proteins 

**Figure 2:**
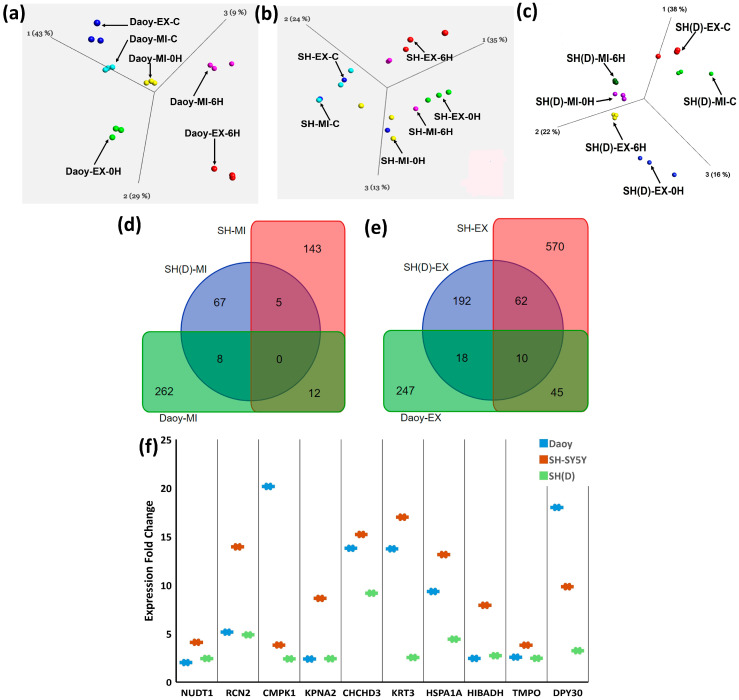
Shared and unique proteins. Principal component analysis (PCA) plots highlighting heat stress conditions and the stress response time point-specific proteins in (**a**) Daoy, (**b**) SH-SY5Y, and (**c**) differentiated SH-SY5Y neuron-like cells. A clear separation is visible according to the stress condition and stress response time point. The input data for PCA plots are available in Supplementary Table S2. Venn diagrams are used to identify the intercellular stress condition-specific proteins. (**d**) No common proteins are identified as part of the response to mild stress. (**e**) Ten proteins are part of the response to extreme stress. The Venn diagram area is not correlated with the number of proteins. (**f**) A whisker plot displaying the 10 DEPs common to the three cell types.

The extreme and mild heat stress response common protein datasets were compared through principal component analysis (PCA) (Supplementary Table S2). The PCA plots constructed for Daoy (Figure 2a), SH-SY5Y (Figure 2b), and SH(D) (Figure 2c) show a clear separation between both the heat stress conditions and the stress response time points. We then analyzed the DEPs based on cell type and stress conditions for their commonality and uniqueness. 

A total of 91 proteins are common to Daoy-EX and Daoy-MI (Supplementary Figure S2a), 65 to SH-SY5Y-EX and SH-SY5Y-MI (Supplementary Figure S2b), and 54 proteins are common between SH(D)-EX and SH-(D)-MI (Supplementary Figure S2c). No proteins common to the three cell types were detected as part of the mild heat stress response, but five are common to SH-SY5Y-EX and SH(D)-EX (Figure 2d). A very high number of 62 proteins were found in SH-SY5Y-EX and SH(D)-EX, potentially correlated with SH-SY5Y being precursors for SH(D)-EX (Figure 2e). There are 58 proteins unique to SH(D)-MI (Figure 2d) and 192 to SH(D)-EX (Figure 2e).

Ten proteins are common to stress response under extreme heat stress (Figure 2f): Cleavage and polyadenylation specificity factor subunit 5 (NUDT21), Reticulocalbin-2 (RCN2), UMP-CMP kinase (CMPK1), Importin subunit alpha-1 (KPNA2), MICOS complex subunit MIC19 (CHCHD3), Keratin, type II cytoskeletal 3 (KRT3), 3-hydroxyisobutyrate dehydrogenase (HIBADH), Lamina-associated polypeptide 2 (TMPO), Protein dpy-30 homolog (DPY30), and Heat shock protein family A member 1A (HSPA1A). A list of proteome-wide common and unique proteins is available in Supplementary Materials Table S2.

### 2.3. Temporal Proteome Analysis

In the global data analysis, we can visualize the time-dependent expression profiles (Figure 1c–e). We further analyzed the temporal behavior of stress response differential protein expression by pairwise t-test comparisons for the three cell types in both stress conditions, thus analyzing Control vs. 0H and Control vs. 6H. 0H is immediately after the stress exposure. 6H post-recovery at 37 °C was selected based on our previous analysis, where chaperone and co-chaperone expressions were observed peaking significantly at 6 h, hence a vital stress response time point [23]. The differential protein expression has a statistically significant difference at *p*-value ≤ 0.05 in the expression ratio. Based on this criterion, in Daoy and SH-SY5Y cells, 5–7% and 10–12% proteomes vary differentially in mild and extreme heat stress, respectively. The neurons represented by differentiated SH-SY5Y neuron-like cells show different behavior with around 25% proteome-wide changes and little difference between extreme and mild heat stress responses. 

**Figure 3:**
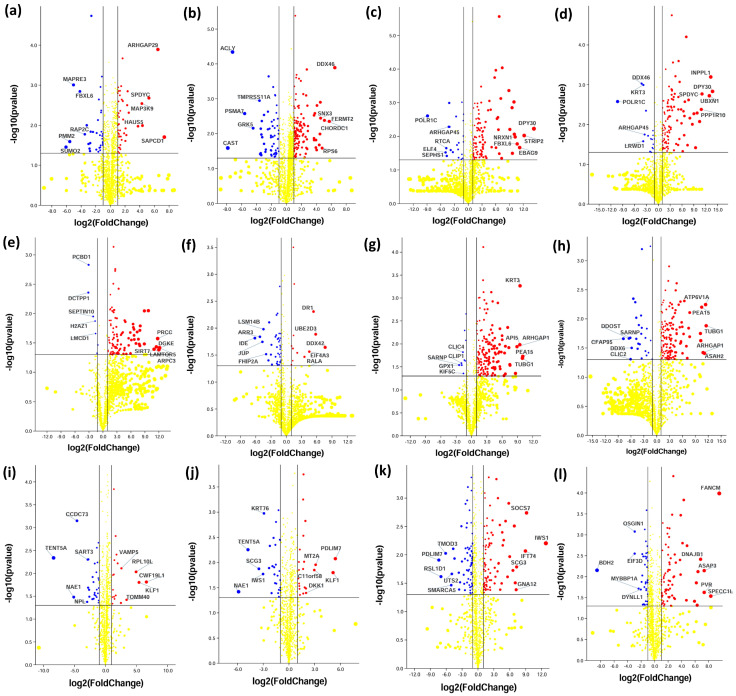
Volcano plots of DEPs that plot log2 fold change on the x-axis and −log10 (*p* value) on the y-axis. Daoy cells (**a**) MI, Control vs. 0H; (**b**) MI, Control vs. 6H; (**c**) EX, Control vs. 0H; (**d**) EX, Control vs. 6H. SH-SY5Y cells (**e**) MI, Control vs. 0H; (**f**) MI, Control vs. 6H; (**g**) EX, Control vs. 0H; (**h**) EX, Control vs. 6H. Differentiated SH-SY5Y neuron-like cells (**i**) MI, Control vs. 0H; (**j**) MI, Control vs. 6H; (**k**) EX, Control vs. 0H; (**l**) EX, Control vs. 6H. Red dots represent upregulated proteins with log2 (fold change ≥ 1); blue dots represent downregulated proteins with log2 (fold change ≤ −1) with a significant difference in the *p*-value at −log10 *p*-value > 1.3. The yellow dots indicate significance by *p*-value but do not reach the fold change threshold for up- and downregulation.

Next, we analyzed the time-series DEPs for up- and downregulation. Proteins expressing with log2 (fold change) ≤ −1 and ≥1 are down- and upregulated, respectively. The results were visualized through volcano plots (Figure 3) that plot log2 fold change on the x-axis and −log10 (*p* value) on the y-axis. Overall, the number of upregulated proteins is mostly higher than that of downregulated proteins. However, a pertinent observation is an intercellular difference in protein up- and downregulation. 

**Figure 4:**
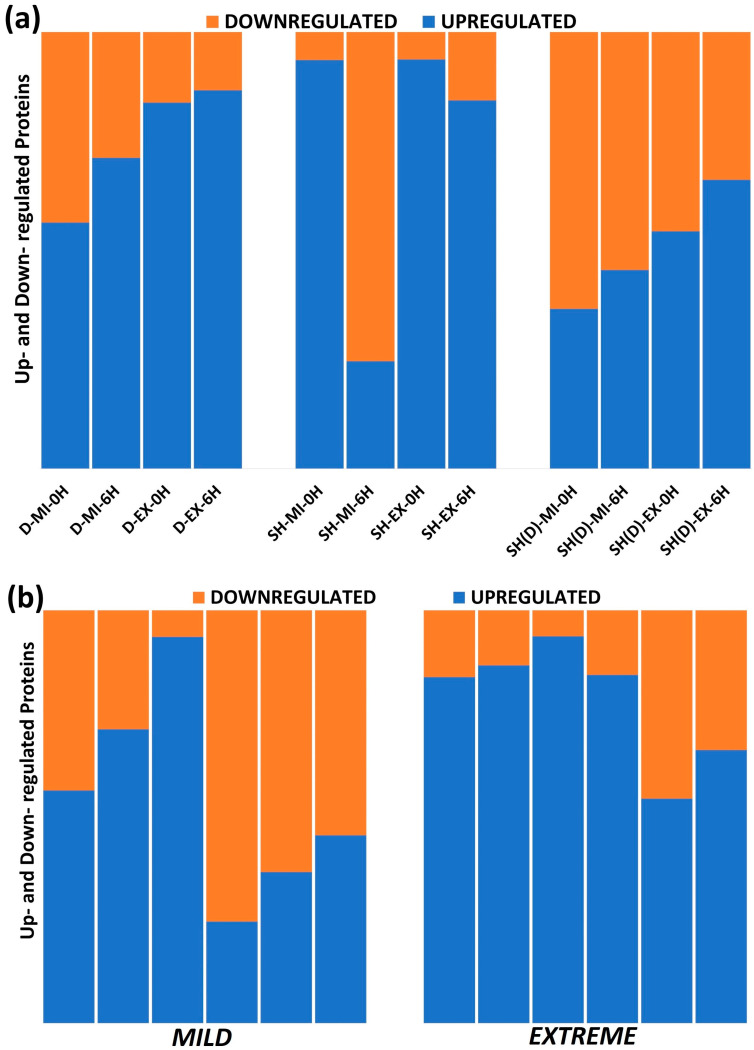

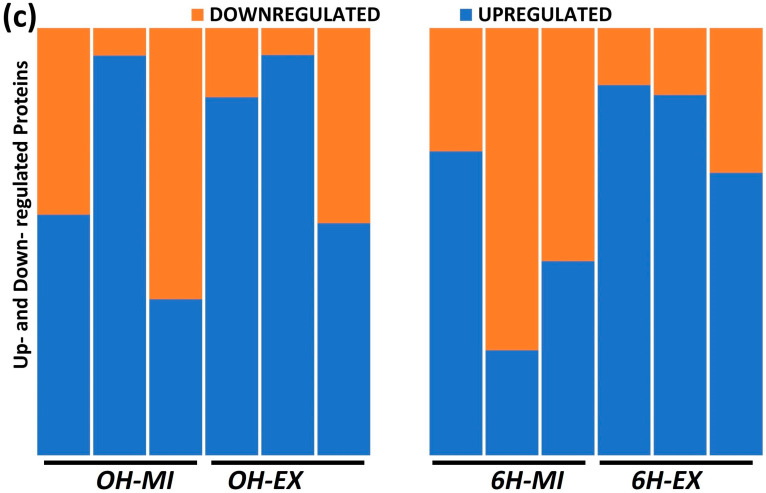
Stacked plot representing the stress condition and response time point-specific up- and downregulated protein numbers. (**a**) Cell-specific numbers: In differentiated SH-SY5Y [SH(D)], compared to Daoy and SH-SY5Y, fewer proteins are upregulated in mild and extreme heat stress conditions. Comparable up- and downregulation levels are also visible in these differentiated neuron-like cells. (**b**) Stress condition-specific numbers: Prominent proteome upregulation is detectable in extreme heat stress. (**c**) Time point-specific numbers: A clearcut stress response time point upregulation pattern is not identifiable, with condition-specific upregulation being the prominent one.

A noteworthy feature is the relatively different proteome mobility in terminally differentiated neuron-like cells. Compared to Daoy and SH-SY5Y, fewer proteins are upregulated in mild and extreme heat stress conditions (Figure 4a). Another significant point is the comparable up- and downregulation levels in these differentiated neuronal-like cells (Figure 4a). Compared to mild stress, remarkably more protein upregulation is observed in response to extreme stress. This is well visualized in the stacked column plot (Figure 4b, right column). Around 65% of the DEPs in Daoy, 75% in SH-SY5Y, and 55% in differentiated SH-SY5Y cells are upregulated in response to extreme heat stress. A time-series analysis of the upregulation does not highlight any time point-specific pattern (Figure 4c). Even in this plot, more upregulation is identifiable in extreme stress response. This observed high upregulation in extreme stress in time-series analysis here coincides very well with the upregulation pattern observed through the global analysis (Figure 1h). This stress response-related differential upregulation highlights the requirement for multi-stress dose and multi-stress response time point analysis in developing a comprehensive understanding of the heat-induced neuronal proteotoxic stress response.

### 2.4. Temporal Proteome Characteristics

#### 2.4.1. Intracellular Temporal Proteome Specificity

**Figure 5:**
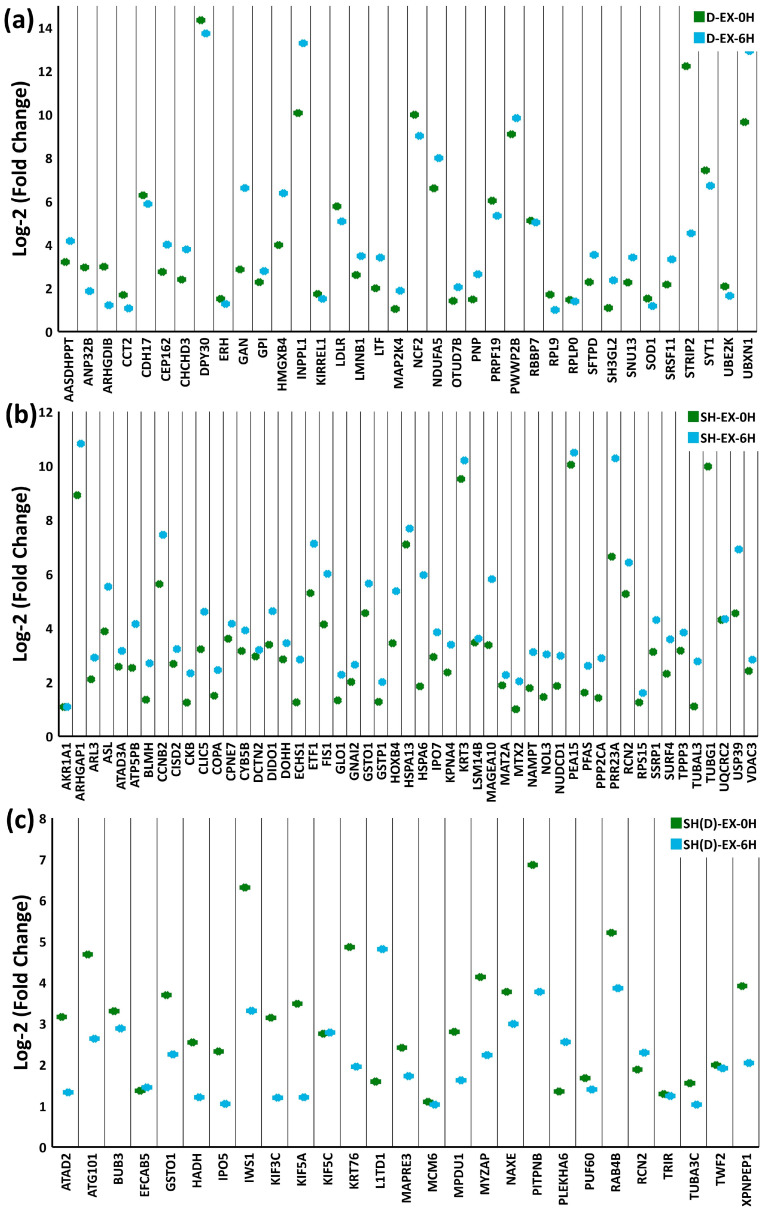
Intracellular extreme condition stress response recovery time point (EX-0H and EX-6H) comparison. (**a**) A total of 36 common proteins upregulated in Daoy; (**b**) 51 in SH-SY5Y; and (**c**) 27 in differentiated SH-SY5Y neuron-like cells. (Venn diagram analysis is visualized in Supplementary Figure S3).

We employed Venn diagrams to discern the unique and common time-specific upregulated stress response proteins. The individual intracellular comparison identified the highest overlapping overexpressed proteins between 0H and 6H in extreme heat stress. In Daoy cells, 36 common proteins are upregulated at 0H and 6H (Figure 5a, Supplementary Figure S3a), while 66 are unique at 0H and 56 at 6H. In SH-SY5Y cells, 51 upregulated proteins are common at 0H and 6H (Figure 5b, Supplementary Figure S3b), while 97 are unique at 0H and 73 at 6H. 

In the differentiated SH-SY5Y neuron-like cells, 27 same upregulated proteins are expressed at 0H and 6H (Figure 5c, Supplementary Figure S3c, Supplementary Table S4). Nine proteins are common to MI-0H and MI-6H: Barrier-to-autointegration factor (BANF1), Non-histone chromosomal protein HMG-17 (HMGN2), Krueppel-like factor 1 (KLF1), Pre-mRNA-processing factor 19 (PRPF19), Ribonuclease kappa (RNASEK), 60S ribosomal protein L13a (RPL13A), Methylcytosine dioxygenase TET3 (TET3), and Vesicle-associated membrane protein 5 (VAMP5) (Supplementary Figures S3c and S4). 

#### 2.4.2. Intercellular Temporal Proteome Specificity

In the intercellular comparison, somewhat surprisingly, not many proteins are common in all three cell types. EX-0H has a single common protein, glutathione S-transferase omega 1(GSTO1) (Supplementary Figure S5a). Only two common proteins are found in EX-6H (Supplementary Figure S5b): the highly homologous Hsp70 family members (HSPA1A) and the ER calcium-binding protein Reticulocalbin-2 (RCN2).

**Figure 6:**
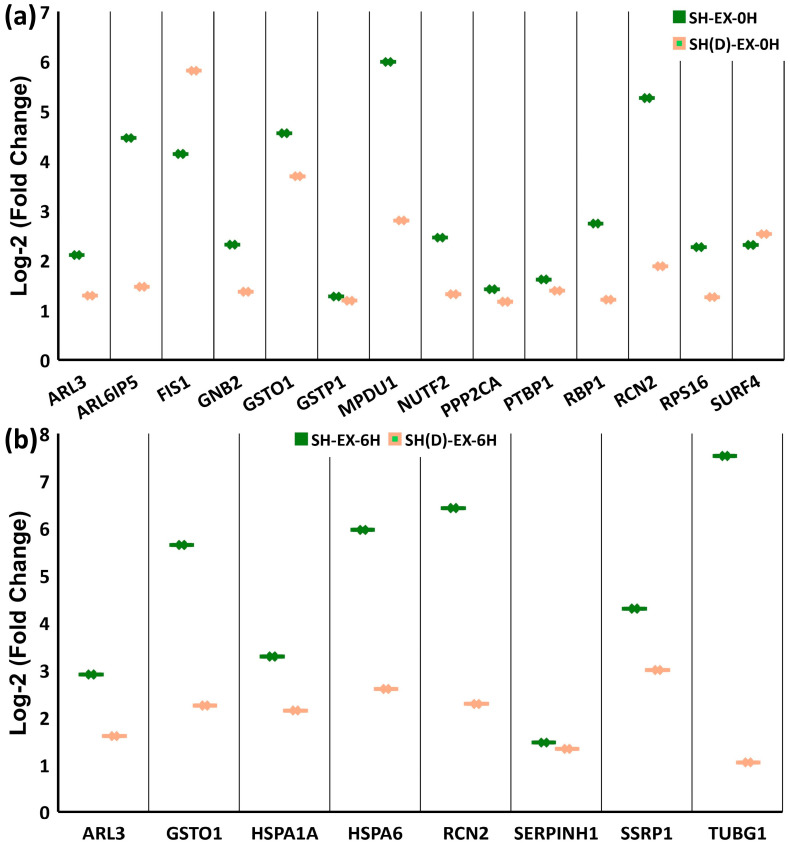
Intercellular extreme condition stress response recovery time point (EX-0H and EX-6H) comparison. (**a**) At EX-0H, fourteen upregulated proteins are expressed in SH-SY5Y and differentiated SH-SY5Y neuron-like cells. (**b**) Eight common proteins between SH-SY5Y and differentiated SH-SY5Y neuron-like cells are upregulated at EX-6H. (Venn diagram analysis is visualized in Supplementary Figure S6).

Expectedly, a relatively higher overlap is observed between SH-SY5Y and the neuron-like differentiated SH-SY5Y neuron-like cells. At EX-0H, in addition to GSTO1, thirteen proteins are common: ADP-ribosylation factor-like protein 3 (ARL3), PRA1 family protein 3 (ARL6IP5), Mitochondrial fission 1 protein (FIS1), Guanine nucleotide-binding protein G(I)/G(S)/G(T) subunit beta-2 (GNB2), Glutathione S-transferase P (GSTP1), Mannose-P-dolichol utilization defect 1 protein (MPDU1), Nuclear transport factor 2 (NUTF2), Serine/threonine protein phosphatase 2A catalytic subunit alpha isoform (PPP2CA), Polypyrimidine tract-binding protein 1 (PTBP1), Retinol-binding protein 1 (RBP1), Reticulocalbin-2 (RCN2), Ribosomal protein S16 (RPS16), and Surfeit locus protein 4 (SURF4) (Figure 6a, Supplementary Figure S6a). Likewise, at EX-6H, six additional proteins are common: ADP-ribosylation factor-like protein 3 (ARL3), Glutathione S-transferase omega 1(GSTO1), Heat shock 70 kDa protein 6 (HSPA6), Serpin H1 (SERPINH1), FACT complex subunit SSRP1 (SSRP1), and Tubulin gamma-1 chain (TUBG1) (Figure 6b, Supplementary Figure S6b).

Unlike Daoy and SH-SY5Y cells, differentiated SH-SY5Y neuron-like cells harbor 83 unique proteins overexpressed at EX-0H (Supplementary Figure S5a) and 70 at EX-6H (Supplementary Figure S5b). Table 2 summarizes cell-wise condition-specific proteins. Supplementary Table S5 shows a more detailed list of all the unique and common proteins. 

#### 2.4.3. Neuronal Recovery Time Point-Specific Protein Characteristics

**Figure 7:**
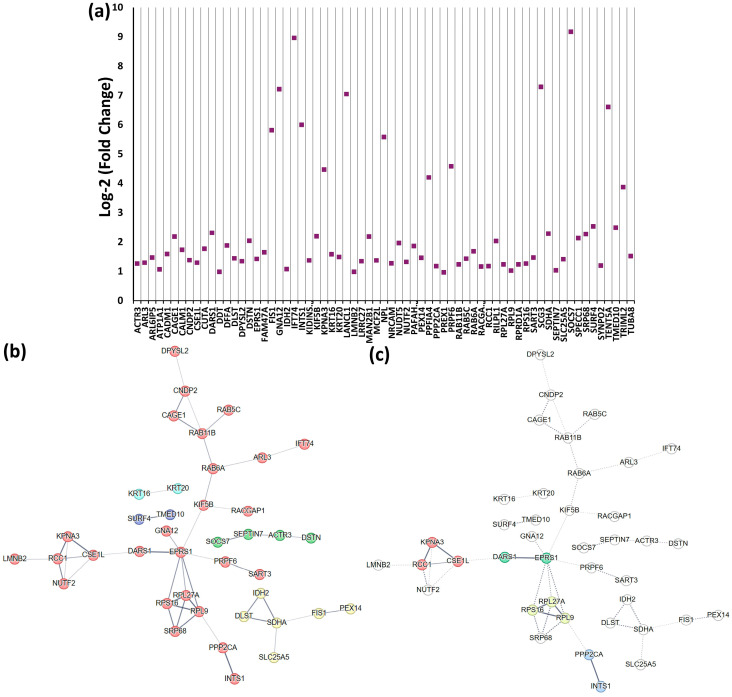

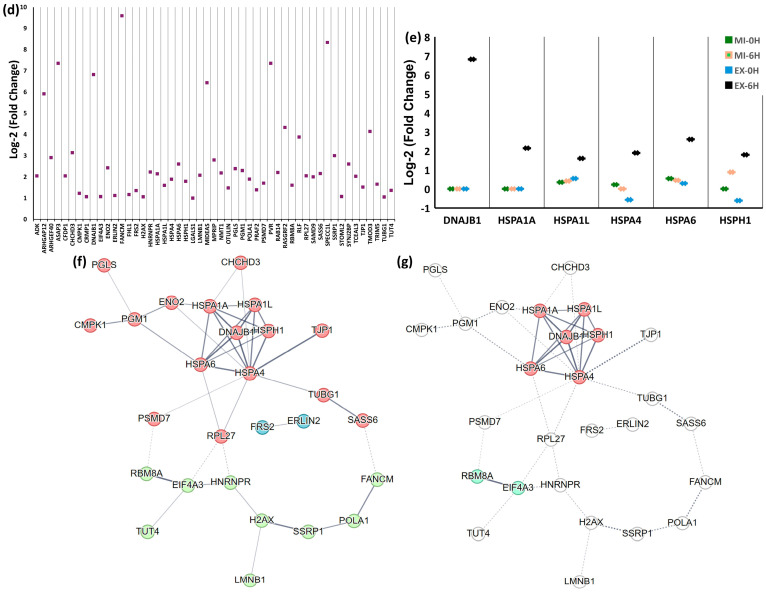
Neuronal (differentiated SH-SY5Y neuron-like cells) extreme stress response recovery time-specific unique protein characteristics. (**a**) Scatter plot for the 67 unique upregulated proteins at EX-0H cells. According to GO analysis, they are engaged in the biological processes of protein localization and transport. (**b**) A k-means (centroid-based) clustering plot of the STRING-based PPI network with the EPRS1-centered cluster (Red) is the most prominent among the five clusters. (**c**) The DBSCAN (node density-based) of the same network identifies 4 subclusters with 2 to 3 members. (**d**) Scatter plot for the 51 unique upregulated proteins at EX-6H in differentiated SH-SY5Y neuron-like cells that, according to gene ontology, are engaged in the biological processes of Unfolded Protein Response and Chaperone cofactor-dependent protein refolding. (**e**) Whisker plot displaying the comparison of EX-0H upregulated chaperones. Although these chaperones are also upregulated in some of the other analyzed conditions, their peak overexpression is observed at EX-6H. (**f**) The k-means clustering of the PPI network identifies the chaperone-containing HSPA4-centered cluster (Red) as the most prominent cluster. (**g**) The chaperone-containing cluster (RED) is the major cluster among the two identified utilizing the DBSCAN mode. In the STRING analysis, the line thickness indicates the strength of data support.

In the differentiated SH-SY5Y neuron-like cells, 67 unique proteins are represented at EX-0H (Figure 7a, Supplementary Table S6) and 51 proteins at EX-6H (Figure 7d, Supplementary Table S7). We constructed an interaction network of these recovery time point-specific proteins using the STRING tool (REF). STRING employs various data sources like text mining, co-expression, neighborhood, gene fusion, and co-occurrence to generate the protein–protein interaction (PPI) network. A k-means clustering, which is a centroid-based clustering, identifies 5 clusters with 26 members in cluster 1 centered around EPRS1, a bifunctional glutamate/proline—tRNA ligase (Figure 7b). On the other hand, a DBSCAN visualization of the EX-0H unique upregulated protein network detects four -two-three membered clusters in this network (Figure 7c). DBSCAN performs a density-based clustering analysis identifying clusters related to node density; thus, clusters are based on the relation closeness among the activity nodes. The gene ontology analysis reveals their involvement primarily in the biological processes of protein localization and transport, impacting the molecular function of protein binding and cytoskeletal protein binding.

Among the fifty-one EX-6H unique proteins (Figure 7d), a remarkable protein group is the chaperone family members HSPA1A, HSPA1L, HSPA6, HSPA4, HSPH1, and DNAJB1 (Figure 7e). In a previous study targeting the chaperone expression in these models, we observed the significant upregulation of some of these chaperones at six hours [23]. k-means clustering identifies a major cluster of sixteen members that harbors all of these chaperone members (Figure 7f). The DBSCAN clustering of this network detects two clusters: one, composed of these chaperone members, and two, clustering Eukaryotic initiation factor 4A-III (EIF4A3) and RNA-binding protein 8A (RBM8A) (Figure 7g). Related to this chaperone upregulation is the detection of biological processes in gene ontology analysis: Unfolded protein response, Chaperone cofactor-dependent protein refolding, and the Nucleoside triphosphate metabolic process related to the molecular functions of ATP-dependent protein folding. Thus, it is fair to speculate that one of the strongest elements of the stress response to heat-induced proteotoxic stress is the protein refolding process. A detailed pathway and network analysis with the DEPs in the three cell types is presented in the following sections. 

### 2.5. Pathway and Functional Analysis

The stress response-specific protein expression datasets identified above were analyzed through ingenuity pathway analysis (IPA) software (www.qiagen.com/ingenuity) to characterize the cellular and functional pathway attributes. We performed the IPA on two differentially expressed protein groups: those identified through ANOVA representing the proteome-wide global response (Section 2.1) and those from pairwise t-test comparisons representing time-specific stress responses (Section 2.3). The IPA output ranks pathways based on the *p*-value. 

#### 2.5.1. Cell-Specific Pathways

**Figure 8:**
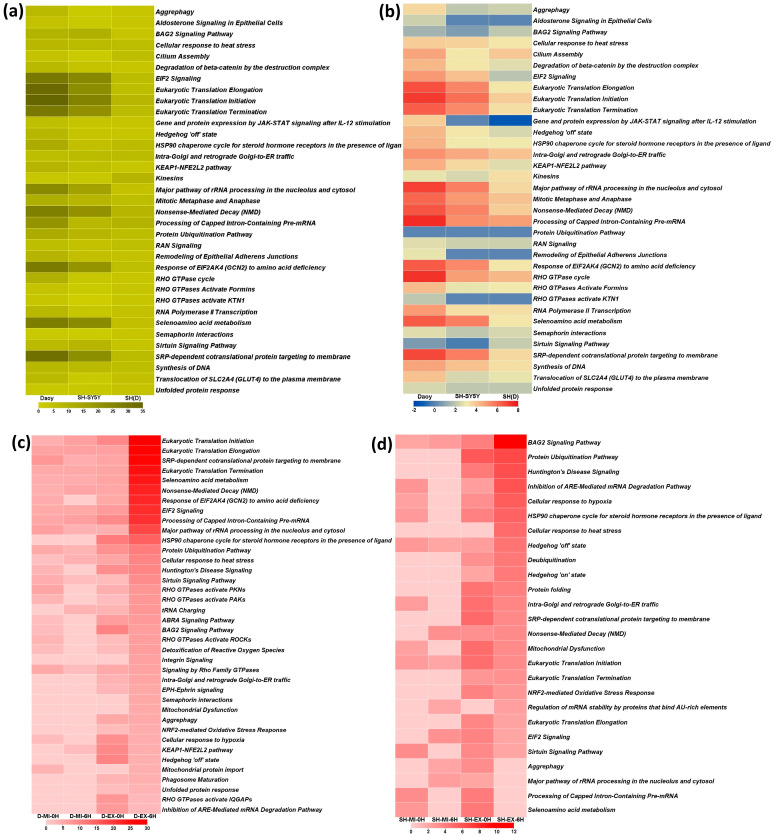

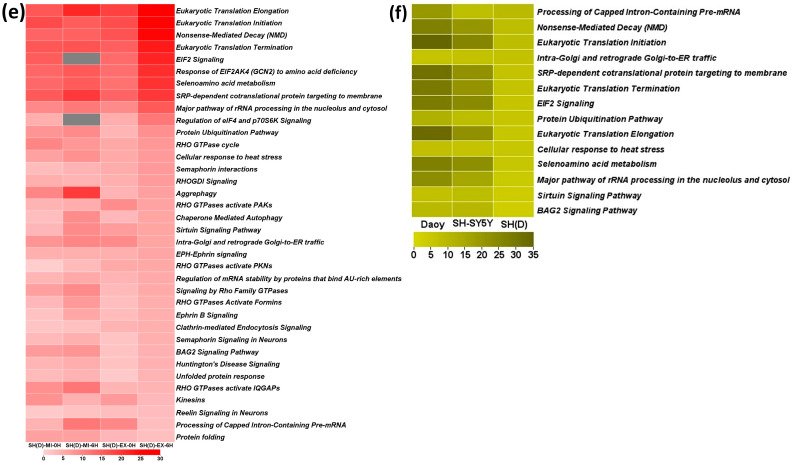
Canonical pathways identified through IPA. (**a**) A comparison of neuron-like differentiated SH-SY5Y [SH(D)]-associated pathways (*p*-value cut-off ≤ 0.0005) with Daoy and SH-SY5Y. Thirty-six pathways are functionally associated with DEPs in neuron-like differentiated SH-SY5Y neuron-like cells. (**b**) The activation z-score profile for the same pathways as in (**a**). The differential activation of these pathways in a cell-dependent manner is visualized. (**c**) A total of 38 pathways were associated with the time-series comparison of the stress response in Daoy. (**d**) A total of 26 pathways were associated with the time-series comparison of the stress response. (**e**) A total of 36 pathways were associated with the time-series comparison of the stress response. (**f**) Comparative pathway analysis identified 14 highly significant (cut-off ≤ 0.00005) common intercellular stress response pathways associated with the heat-induced proteotoxic stress response. [Heat maps a, c, d, e, and f are based on −Log 10 (*p*-value)].

At the association cut-off *p*-value ≤ 0.0005, 56, 39, and 36, canonical pathways are associated with the response to heat-induced proteotoxic stress in Daoy, SH-SY5Y, and differentiated SH-SY5Y neuron-like cells, respectively (Supplementary Figure S7). Comparing the differentiated SH-SY5Y neuron-like cell-associated pathways with the other two cell types reveals their differential involvement (Figure 8a). In addition to the association *p*-value, the IPA computes another statistical parameter called the activation z-core, an IPA Downstream Effects analytic. The Z-score profile also highlights the differential activation of these pathways in a cell-dependent manner (Figure 8b). In the time-series comparison of the stress response to extreme and mild heat, 38 canonical pathways are identified for Daoy (Figure 8c), 26 for SH-SY5Y (Figure 8d), and 36 for differentiated SH-SY5Y neuron-like cells (Figure 8e).

In the Daoy cells, the top five pathways that are represented globally and also display time-specific behavior include Eukaryotic Translation Initiation, Eukaryotic Translation Elongation, Eukaryotic Translation Termination, EIF2 Signaling, and SRP-dependent cotranslational protein targeting to membrane. On the other hand, Aggrephagy, NRF2-mediated oxidative stress response, Hedgehog off state, Phagosome maturation, and Unfolded Protein Response are specific only to extreme heat stress (Figure 8c). At the same time, integrin signaling, semaphorin interactions, and mitochondrial dysfunction are part of the response to extreme heat stress after 6 h of stress exposure (Figure 8c).

In SH-SY5Y cells, the same pathways are not represented among the top five in two differently analyzed protein datasets. Eukaryotic Translation Initiation, Eukaryotic Translation Elongation, EIF2 Signaling, and SRP-dependent cotranslational protein are among the top five pathways, with Eukaryotic Translation Termination ranking sixth in the overall response to heat stress. In contrast, in the time-series analysis, the top five ranking pathways include the BAG2 Signaling Pathway, Protein Ubiquitination Pathway, Huntington’s Disease Signaling, Inhibition of the ARE-mediated mRNA decay pathway, and Cellular response to Hypoxia (Figure 8d). A notable pathway is the Cellular response to heat stress pathway, which is only associated with an extreme heat stress response 6 *h* into recovery (Figure 8d).

Differentiated SH-SY5Y neuron-like cells exhibit a behavior similar to that of SH-SY5Y cells, and different pathways represent the two groups analyzed. The Processing of Capped Intron-Containing Pre-mRNA, Kinesins, Nonsense- Mediated Decay (NMD), Eukaryotic Translation Initiation, and Hsp90 chaperone cycle for steroid hormone receptors in the presence of ligand pathways represent the overall stress response. At the same time, Eukaryotic Translation Elongation, Eukaryotic Translation Initiation, Nonsense- Mediated Decay (NMD), Eukaryotic Translation Termination, and EIF2 Signaling are the prominent pathways in the time-series analysis (Figure 8e). A notable highlight in differentiated SH-SY5Y neuron-like cells compared to the Daoy and SH-SY5Y cells is that the associated pathways are universally correlated with different heat stress response conditions and response time points (Figure 8e). 

#### 2.5.2. Highly Significant Common Pathways

We undertook a multi-pronged Venn diagram-based approach to identify highly significant common intercellular pathways. We considered pathways with the association cut-off *p*-value ≤ 0.00005. In the first approach, the initial step involved the intercellular comparison between the overall stress response pathways (pathway analysis of ANOVA results), identifying 20 pathways common to the three cell types (Supplementary Figure S8a). A similar intercellular comparison between the time-dependent stress response pathways identified 16 common pathways (Supplementary Figure S8b). Next, we looked for the common pathways between these two analyses and identified 14 stress response pathways (Supplementary Figure S8c). The next approach involved the intracellular comparison between pathways identified based on a holistic analysis (ANOVA) of the stress response and pathways associated with time-dependent response. This identified 35, 23, and 21 common pathways in Daoy, SH-SY5Y, and differentiated SH-SY5Y neuron-like cells (Supplementary Figure S9a–c). Like in the first approach, we searched for the common pathways among these common pathways and identified 14 stress response common pathways (Supplementary Figure S9d).

The 14 stress response pathways identified through the two approaches are similar. Thus, 14 of the canonical pathways expressed as part of heat-induced proteotoxic stress are common to differentiated neuron-like cells and other associated cell types (Figure 8f). These pathways are functionally associated with the biological processes of protein, RNA and cellular metabolism, Cellular Response to Stress, Membrane Trafficking, and Cell Signaling, thereby mainly impacting the molecular and cellular functions of Gene Expression, Cellular Function and Maintenance, Cell Death and Survival, Post-Translational Modification, Protein Folding, and Protein Synthesis [25,26]. Cellular response to heat stress involves rapid but transient transcriptional-level molecular reprogramming to adjust to the changing cellular circumstances by modifying processes like RNA and protein synthesis, cellular metabolism, and cytoskeletal and membrane integrity [27,28,29]. Thus, associating 14 commonly identified pathways and their related biological processes with the heat-induced stress response is a natural consequence of the cellular requirements and consequent stress response regulation. 

#### 2.5.3. Biological Functions and Disease

The functional aspect of IPA enables associating biological functions and diseases with the experimental outcomes of protein expression changes. There are a total of 26, 7, and 15 Molecular and Cellular Functions associated in Daoy, SH-SY5Y, and differentiated SH-SY5Y neuron-like cells, respectively (Supplementary Figure S10). The six functions, Gene Expression, Cellular Function and Maintenance, Cell Death and Survival, Post-Translational Modification, p Protein Folding, and Protein synthesis found in SH-SY5Y are common to the three cell types. In differentiated SH-SY5Y neuron-like cells, only two pathways, Cellular Function and Maintenance and Protein Synthesis from the top five are active at 0H and 6H in mild and extreme stress responses (Supplementary Figure S11). 

In the differentiated SH-SY5Y neuron-like cells, 6, 5, 11, and 17 pathways of the Physiological System Development and Function are significantly associated with MI-0H, MI-6H, EX-0H, and EX-6H, respectively (Supplementary Figure S12). Three joint Physiological System Development and Function pathways are used in the four groups: Tissue Development, Organismal Survival, and Organismal Development. The analysis of the top five pathways (Supplementary Figure S13) reveals Organismal Survival as a single common pathway. Nervous System Development and Function and Endocrine System Development and Function are associated with EX-0H, EX-6H, and MI-6H, apparently correlated to the enhanced stress response compared to MI-0H. Among Diseases and Disorders (Supplementary Figure S14), Neurological Disease, Organismal Injury and Abnormalities, Hereditary Disorder, Psychological Disorders, and Skeletal and Muscular Disorders are particularly interesting in the context of neuronal stress (Supplementary Figure S15). The two disorders Neurological Disease and Organismal Injury and Abnormalities are associated with all four analyzed conditions. 

### 2.6. Neuronal Temporal Pathway Analysis

**Figure 9:**
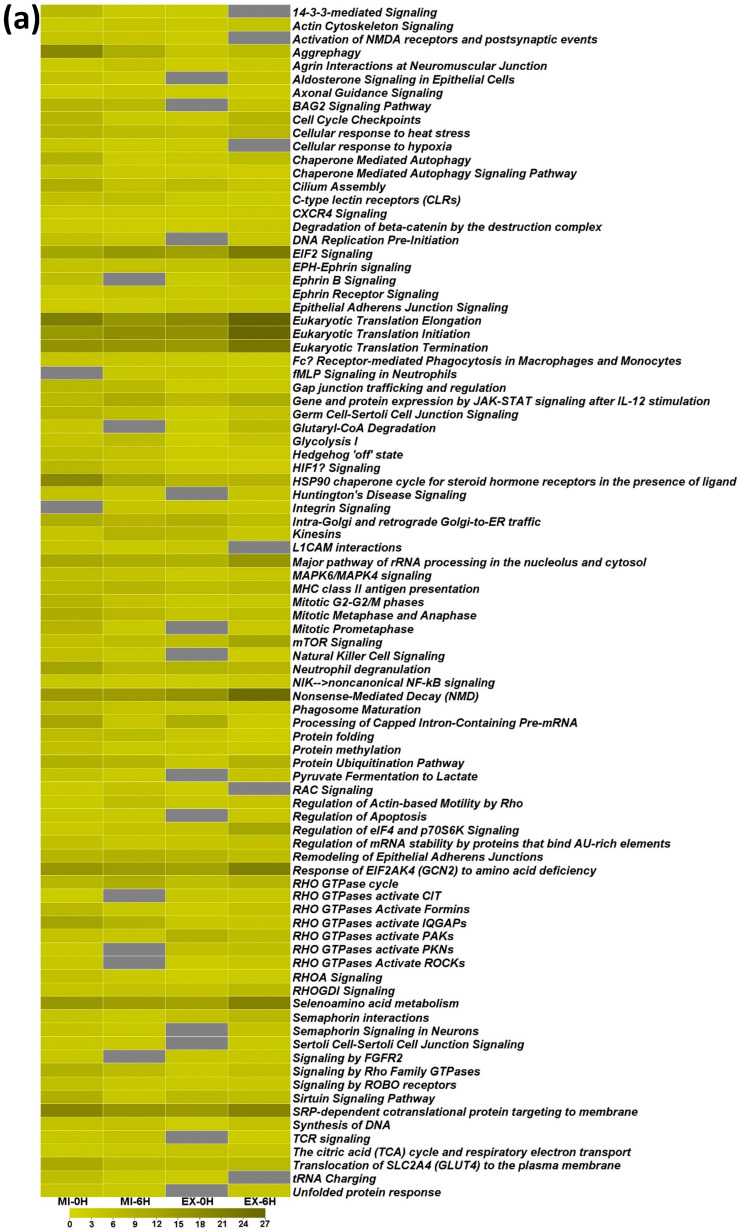

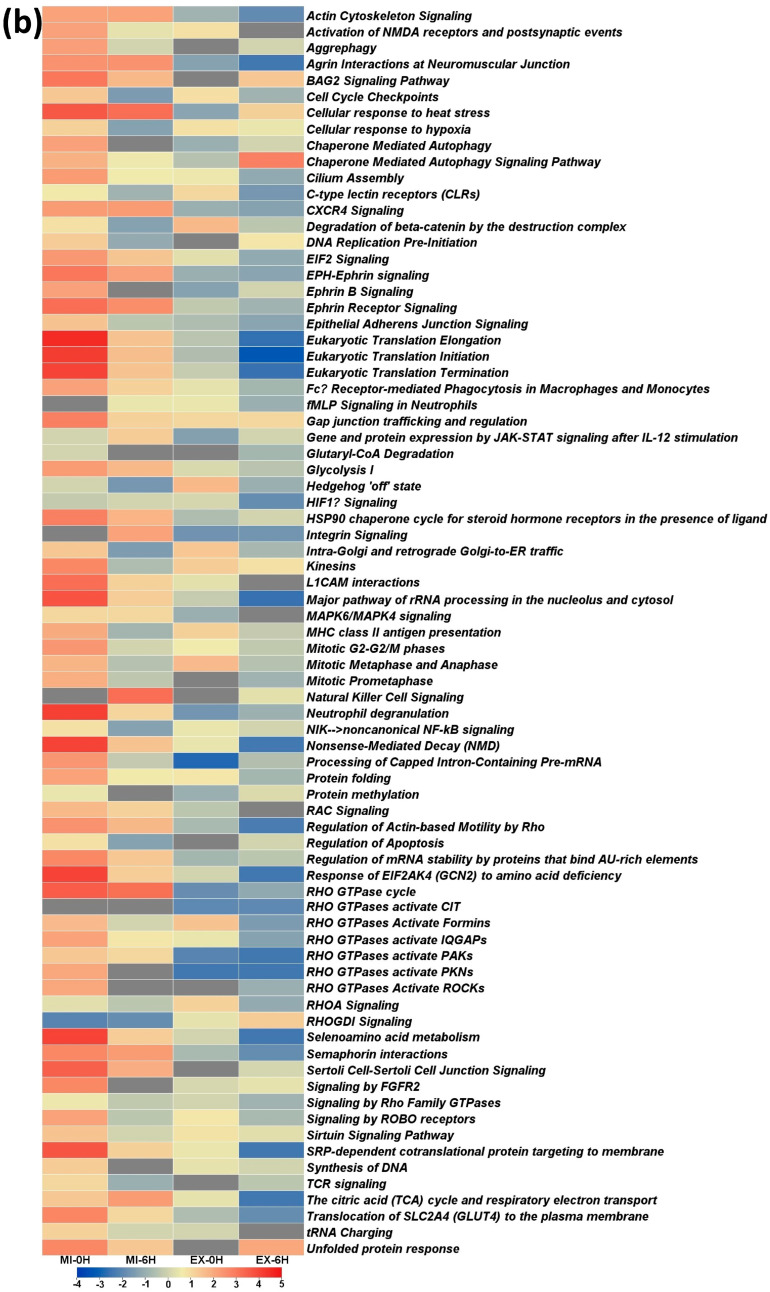
Neuronal temporal pathway analysis. (**a**) Eighty-nine functional pathways at association *p*-value ≤ 0.005 identified in differentiated SH-SY5Y neuron-like cells in MI-0H, MI-6H, EX-0H, and EX-6H. Expectedly, the pathways display a differential association with the analyzed conditions. (**b**) The heat map for IPA Downstream Effects analytic, activation z-score for 77 associated pathways highlights the differential engagement of the common functional pathways with different analyzed conditions.

We undertook a detailed temporal analysis of the significantly associated pathways in differentiated SH-SY5Y neuron-like cells. We considered pathways with the cut-off for association *p*-value ≤ 0.005 or a −Log(*p*-value) of 2.3 for further analyses. According to this criterion, 162, 109, 105, and 137 pathways are associated with MI-0H, MI-6H, EX-0H, and EX-6H. After considering the common pathways, 211 pathways show a temporal association with the stress response. The inclusion criterion for a pathway’s time-series analysis was that it be present in at least three condition–time point combinations. Ninety pathways meet this criterion, Supplementary Table S8, and after excluding the Coronavirus Pathogenesis Pathway, 89 pathways were further analyzed. Fifty-nine pathways are common to all four conditions (Supplementary Figure S16). As expected, the 89 associated pathways display a differential association with different analyzed conditions, as reflected in their p-values (Figure 9a). Out of 89 pathways, z-scores were available for only 77 pathways, which means directionality was not reliably measurable in the other 12 pathways. Z-score profiles also depict an analyzed point-specific pattern (Figure 9b). Thus, despite the commonality of the functional pathways in the stress response, there is both a stress dose-specific and recovery time point-specific engagement of these functional pathways. 

The 89 pathways identified here are implicated in 15 biological processes: Signaling (34), Protein Metabolism (8), RNA Metabolism (4), Cellular Metabolism (6), Cellular Response to Stress (4), Cellular Response to Heat Stress (2), Autophagy (2), Programmed Cell Death (1), Organelle Biogenesis (1), Cell Cycle (5), Cell Cycle Communication (1), DNA Replication (1), Molecular Transport (4), and Immune Response (8). In addition, seven pathways are associated with neuron-specific biological processes. Thus, the heat-induced proteotoxic stress response in human neuronal cell models is accompanied by the induction of multiple biological processes that have implications for routine cellular activities and stress-specific functions. A detailed explanation regarding the functional implication of these pathways is discussed in Section 3.1, Section 3.2 and Section 3.3.

### 2.7. Biological Networks

Another feature of IPA is the establishment of biological networks for the identified pathways. These networks are composed of interacting proteins characterized by specific and extensive interactions in the ingenuity knowledge base. In these networks, the interacting proteins are represented as nodes, with a line representing a direct biological relationship and a hashed line an indirect one. We performed a detailed protein–protein interaction analysis through STRING to further characterize these identified networks. In addition to a thorough functional evaluation, STRING analysis identified clusters within these networks utilizing k-means clustering that clusters networks based on centroids. We limited our search to the identification of three clusters. 

#### 2.7.1. Intercellular Comparison

A total of 25 protein networks in Daoy (Supplementary Materials Table S9) and SH-SY5Y (Supplementary Materials Table S10) and 27 in differentiated SH-SY5Y (Supplementary Materials Table S11) cells are associated with the differentially abundant proteins in the overall response to heat-induced proteotoxic stress (proteins identified through ANOVA). Of these 20, 18, and 12 networks in Daoy, SH-SY5Y, and differentiated SH-SY5Y neuron-like cells rank with a score ≥ 20.

**Figure 10:**
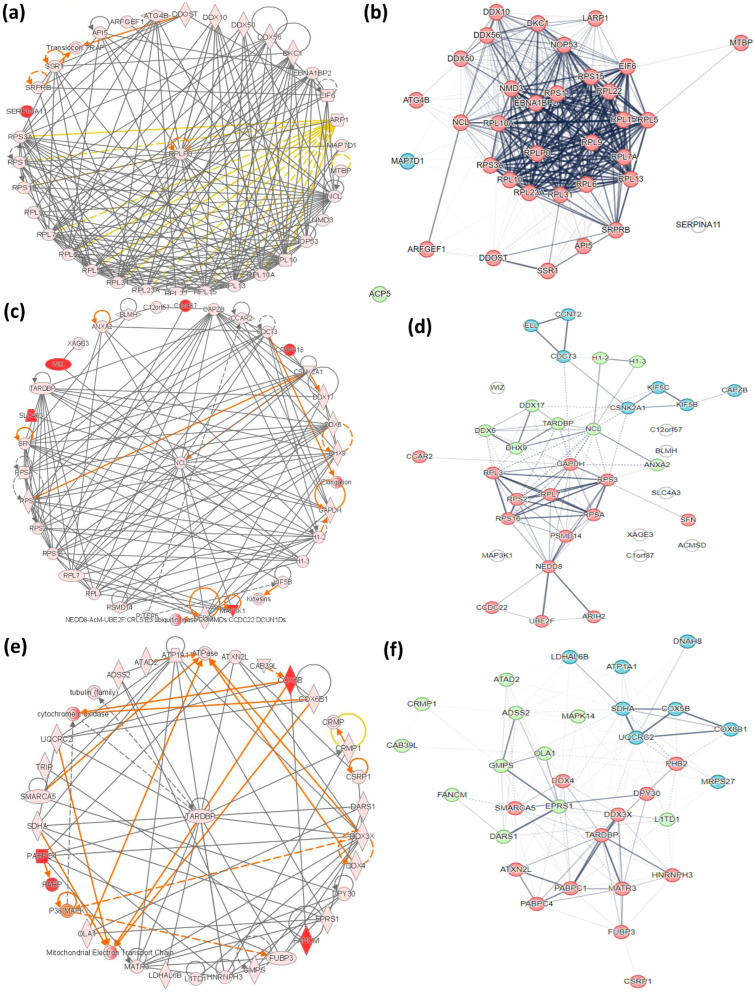
Networks associated with DEPs. Daoy cells (**a**) The IPA identified highest-ranked protein network of functional associations between 34 proteins is related to *Cancer*, *Cell Death and Survival*, *Organismal Injury*, *and Abnormalities* with the central node as RPLP0. (**b**) The RPLP0-centered cluster (Red) is the most prominent among the three clusters identified by STRING analysis. SH-SY5Y cells (**c**) The IPA of the highest-ranked protein network of functional associations between 31 proteins is related to *Cellular Assembly and Organization, Gene Expression, and RNA Post-Transcriptional Modification* with the central node as Nucleolin (NCL). (**d**) In the STRING analysis, Nucleolin (NCL) identified as the central node through IPA is part of the second primary cluster (Green). Differentiated SH-SY5Y neuron-like cells (**e**) The IPA highest-ranked network in the differentiated SH-SY5Y neuron-like cells composed of 28 proteins is related to *Hereditary Disorders, Neurological Diseases, Organ Injury, and Abnormalities* with TARDP as the central node. (**f**) TARDP is part of the first cluster (Red) in the STRING analysis. *(In the IPA networks, the color of the shape indicates the degree of upregulation (red) or suppression (green) with color intensity relative to the expression. The direct biological relationship between two nodes is a line, and an indirect relationship is a hashed line. The arrows indicate the protein-protein interactions).*

In the Daoy cells, the highest-ranked pathway (Score = 54, Focus Molecules = 34) is related to Cancer, Cell Death and Survival, Organismal Injury, and Abnormalities. The central node was identified as 60S acidic ribosomal protein P0 (RPLP0) (Figure 10a). Among the three clusters, the RPLP0-centered cluster (Red) is the prominent one, and the other two clusters (Green and Blue) are mono-molecular (Figure 10b).

In the SH-SY5Y cells, the highest-ranked pathway (Score = 53, Focus Molecules = 31) is associated with Cellular Assembly and Organization, gene expression, and RNA Post-Transcriptional Modification with Nucleolin (NCL) as its central node (Figure 10c). This network is distributed relatively evenly into three clusters, among which NCL (in green), identified as the central node through IPA, is part of the second central cluster (Figure 10d). 

The highest-ranked network in the differentiated SH-SY5Y cells (Score = 51, Focus Molecules = 28) is related to Hereditary Disorders, Neurological Diseases, Organ Injury, and Abnormalities with TAR DNA-binding protein 43 (TARDP), an RNA-binding protein, as the central node (Figure 10e). The protein molecular interactors in the three clusters are evenly distributed, and TARDP, the central node identified in the IPA, is part of the first cluster (Red) (Figure 10f).

#### 2.7.2. Differentiated SH-SY5Y Neuron-Like Cells Time-Series Comparison

**Figure 11:**
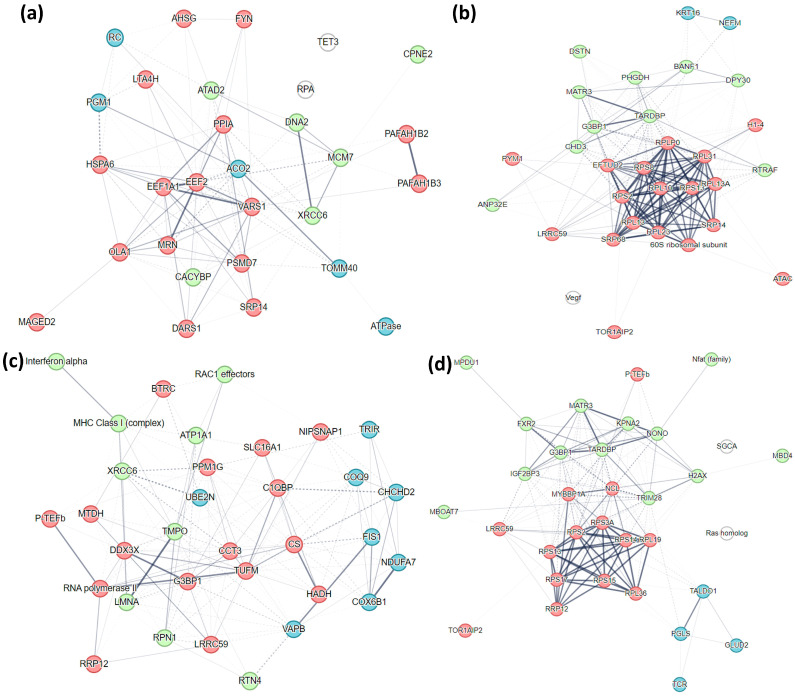
Networks associated with DEPs in differentiated SH-SY5Y neuron-like cells. (**a**) MI-0H: *Cellular Assembly and Organization, Lipid Metabolism, and Small Molecule Biochemistry*, with 25 proteins, is the topmost network in MI-0H, with 16 interactors in Cluster-1 (Red); (**b**) MI-6H: *Cancer, Cell Death, and Survival, Organismal Injury, and Abnormalities*, with 28 proteins, is the high-ranking network, with 18 interactors in Cluster-1 (Red); (**c**) EX-0H: *Cell Morphology, Cellular Assembly and Organization, DNA Replication, Recombination, and Repair,* with 28 proteins, is the most prominent network containing 16 interactors in Cluster-1 (Red); (**d**) EX-6H: *Cellular Assembly and Organization, Cellular Function, and Maintenance, Gene Expression* containing 30 proteins with 14 interactors in main Cluster-1 (Red). *(The line thickness indicates the strength of data support).*

We also performed the network analysis for the pathways associated with differentially abundant proteins in differentiated SH-SY5Y neuron-like cells identified from the pairwise time-series comparison in mild and extreme models. Twenty-five biological networks are associated with stress response in mild and extreme conditions at 0 and 6 h post-stress exposure. Of these, 11, 8, 11, and 14 networks rank with a score ≥ 20 in MI-0H, MI-6H, EX-0H, and EX-6H, respectively (Supplementary Materials Table S12). The topmost (Score = 44, Focus Molecules = 25) network for MI-0H is associated with functions of Cellular Assembly and Organization, Lipid Metabolism, and Small Molecule Biochemistry. Cluster-1 (Red) has 16 interactors, while clusters 2 (Green) and 3 (Blue) have 6 and 5 interactions, respectively (Figure 11a). Cancer, Cell Death and Survival, Organismal Injury, and Abnormalities (Score = 54, Focus Molecules = 28) is the high-ranking network associated with disease and function in MI-6H. The three clusters in this network have 18 (Red), 10 (Green), and 2 (Blue) interactions (Figure 11b). For EX-0H Cell Morphology, Cellular Assembly and Organization, DNA Replication, Recombination, and Repair (Score = 51, Focus Molecules = 28) is the most prominent network with 16 (Red), 9 (Green), and 8 (Blue) interactions in the three clusters (Figure 11c). The prominent network (Score = 57, Focus Molecules = 30) in EX-6H is associated with Cellular Assembly and Organization, Cellular Function and Maintenance, and g Gene Expression clustering to 14 (Red), 13 (Green), and 4 (Blue) interactions (Figure 11d). 

## 3. Discussion

Heat stroke is a life-threatening illness among heat-related maladies with both immediate and long-term CNS malfunctions as a significant detriment [15]. A principal causative agent for these malfunctions is thermal fluctuation-induced protein misfolding and aggregation [28,29]. The neurotoxic behavior of misfolded proteins and their aggregated products is a known fact and, consequently, a causal factor for neurodegenerative disorders [17]. Despite protein aggregation being the connecting link between hyperthermia-induced neurological damage and other neurological damages, the higher rate of aggregate accumulation potentially leads to a specific pathophysiology [13,21,22]. Another unique feature of hyperthermia-induced neurological damage is its dose-responsive nature. 

Here, we present an in vitro proteomics map of human neurons’ heat-induced dose-responsive proteotoxic stress models. These models in medulloblastoma [Daoy], neuroblastoma [SH-SY5Y], and differentiated SH-SY5Y neuron-like cells cover a range of stress conditions [23]. The stress response is transient yet effective in ensuring cell recovery; consequently, to avoid overdoing, the stress response is balanced with stress severity and controlled in magnitude and duration [30]. Expectedly, stress response is a dynamic process with different stages correlated with cell fate outcomes. Thus, time-series proteomic studies in dose-responsive heat stress neuronal models provide an effective tool for mapping the active functionality and developing knowledge about molecular mechanisms engaged in cellular adaptation and survival. Our experimental setup analyzed 18 biological samples from three in vitro neuronal models that represent two stress states, extreme and mild, and two time points in the recovery phase. Cumulatively, we quantitated 2957 proteins across all replicates and time points. This dataset is comparable to datasets utilized in stress response studies [31,32].

As part of the stress response, the number of differentially varying proteins in Daoy and SH-SY5Y is 5–7% and 10–12% of proteomes in mild and extreme stress response. This dose-dependent variation in Daoy and SH-SY5Y is explicable in the context of the cellular requirement to adapt to variable stress states and the magnitude-controlled programming of the stress response pathways [33]. Differentiated neuron-like cells, on the other hand, have a similar proteome-wide change of around 25% during both extreme and mild stress responses. This translates into a total of 809 DEPs across the four condition–time point combinations. Another interesting feature of the differentiated SH-SY5Y neuron-like cells is the comparable up- and downregulation levels in extreme and mild heat stress compared to the other two cell types, where around 50% more proteins express differentially in extreme conditions. The similar proteome change in mild and extreme heat stress in differentiated SH-SY5Y neuron-like cells does not translate into changes in similar proteins. The stress-induced proteome-wide changes involve counterbalancing actions through the member depletion of non-essential housekeeping or pathway-specific proteins [34,35].

The number of common and unique proteins shows the variable proteome engagement during mild and extreme stress responses in differentiated SH-SY5Y neuron-like cells (Section 2.4). In extreme heat stress response, only 19% (27/145) of the upregulated proteins are common to two recovery points, with 46% unique to 0H and 35% uniquely expressed at 6H. The noteworthy point is that even in extreme conditions, the limited number of chaperome members are common to EX-0H and EX-6H. Chaperones and proteases play a dual role in maintaining protein quality control (PQC) and facilitating protein unfolding and disaggregation [36], thus a critical component of proteostasis machinery. Of the 332 chaperome members [37,38], only 23 are differentially expressed at EX-0H and 20 at EX-6H, a relatively small percentage of around 6–7, of which 9 vary differentially in both time points. However, none of the upregulated members are common to the two analyzed time points. At EX-6H, six chaperone members HSPA1A, HSPA1L, HSPA4, HSPA6, HSPH1, and DNAJB1 are upregulated. Similar outcomes about the expression of a limited number of chaperones and co-chaperones in neuronal heat stress are reported [23,39]. Although these results contrast with the higher chaperome engagement reported in other studies [40,41,42,43], it is pertinent to note that these are transcriptomics-based studies conducted in PBMCs isolated from human heat stroke patients. Thus, it is plausible to conclude that neuronal stress response is relatively conservative in the number of molecules and, consequently, very tightly regulated.

The temporal analysis of the significantly associated pathways in differentiated SH-SY5Y neuron-like cells (Section 2.6) provides a deeper insight into the functional role of these pathways during the stress response. The statistical parameter activation z-score infers the activation states (increased or decreased) of the associated pathways and biological functions. It analyzes the pathway-specific protein dataset for its change in direction as defined by the expression pattern in samples when compared to the control. Of the 89 identified pathways, z-scores were computed for 77 pathways (Figure 9). We discuss this analysis in the context of the biological process and associated cellular function. 

### 3.1. Transcription, Translation, and Protein Metabolism

**Figure 12:**
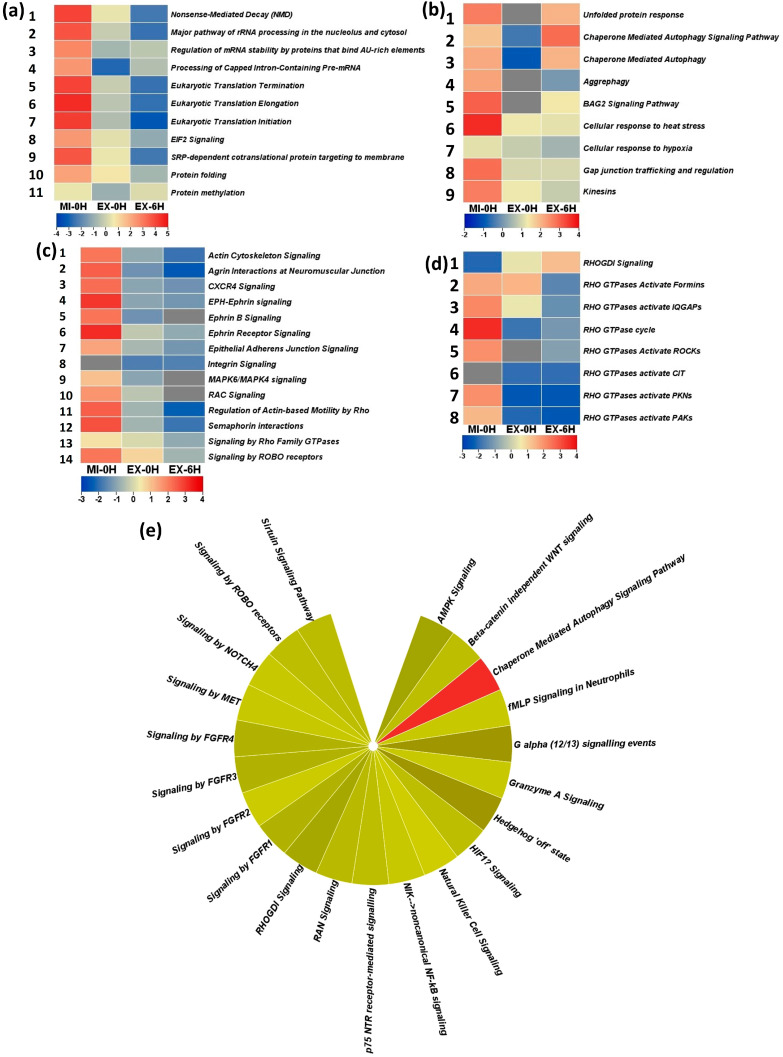
(**a**) Activation z-score heat map for molecular pathways associated with transcription, translation, and protein metabolism. Four pathways are related to RNA metabolism, and seven pathways to protein metabolism. (**b**) Activation z-score for seven protein metabolism-related pathways, two protein trafficking, and two cellular stress response pathways related to Proteostasis Maintenance and cellular stress response molecular functions. (**c**) Signal transduction pathways that are highly activated during response to mild heat stress. (**d**) Differential RHOGD1 Signaling during mild and extreme heat stress. Its inactivation during mild heat stress ensures the activation of RHO GTPases, while it is vice versa during extreme heat stress with active RHOGD1 Signaling to inhibit RHO GTPase activity. (**e**) Twenty-four signaling pathways are differentially activated during response to extreme heat stress.

This group involves four pathways related to the biological process of RNA metabolism and seven pathways related to protein metabolism (Figure 12a). *Nonsense-mediated decay (NMD)* is a regulatory translation quality control pathway that recognizes and eliminates mutant mRNAs with premature termination codons [44,45]. NMD inhibition is a hallmark of cellular stress, a dynamic adaptive regulation correlated with stress response and cellular survival [42]. From this rationale, the activation/inhibition behavior of NMD in the condition–time point analysis is very well explicable: high activation in mild condition (MI-0H) compared to extreme conditions (EX-0H), which further falls at EX-6H. Like NMD, the *Major pathway of rRNA processing in the nucleolus and cytosol* also exhibits a stress-specific inhibition pattern. Ribosomal protein synthesis accounts for a quarter of the total protein synthesis [43]; the inhibition of rRNA processing is a cell survival mechanism that curtails the energy spent on ribosomal protein synthesis [45]. Pre-mRNA processing, including 5′ cap addition, ensures gene expression fidelity and efficiency [46]. The cellular stress response is a conservative process aimed at cell survival; hence, the inhibition of mRNA splicing is such an adaptation observed during heat shock [47]. The highest inhibition in the *Processing of Capped Intron-Containing Pre-mRNA* at EX-0H is attributable to the total shutdown of the RNA processing pathways as an immediate response to heat stress that is subsequently relaxed during the recovery phase (Figure 12a, Block 4). Compared to the previous three RNA processing pathways, the *Regulation of mRNA stability by proteins that bind AU-rich elements* activation is a pro-stress response adaptation. mRNAs with AU-rich elements encode proteins that augment cellular adaptation to challenges like inflammatory stimuli and environmental factors [48,49]. Consequently, the activation of this pathway, even at EX-6H (Figure 12a, Block 3 3), indicates this pathway’s involvement in the heat-induced proteotoxic stress response. The overall inhibition of RNA processing should not be surprising because mRNA quality control bypassing ensures the quicker transfer of mRNA into translation machinery [50]. Additionally, stress response transcription regulation ensures expedited transcription regulation through nucleosome organization and gene structural reorganization [51,52]. Correlated to transcription inhibition is translation arrest, a hallmark of the stress response pathways to modulate the utilization of this energetically expensive process [53,54]. Thus, the repression of translation-associated pathways *Eukaryotic Translation Initiation*, *Eukaryotic Translation Elongation*, and *Eukaryotic Translation Termination* (Figure 12a, blocks 5–7) is not surprising. Therefore, the relatively constant engagement of *eIF2 Signaling* is related to translation repression. Translation initiation is a rate-limiting step; hence, the regulation of this step is critical to translation inhibition [53]. Among multiple stress response mechanisms of translation inhibition, a commonly used mechanism is the phosphorylation of the translation initiation factor eIF2 [53,55]. This occurs through stress and physiological stimuli-sensitive factor-specific signal transduction pathways [54]. eIF2 phosphorylation has a nonsense-mediated decay (NMD) repressive function that, in turn, has a role in stabilizing and expressing stress response factors [42]. Considering these pathways’ activation/inhibition patterns and interfunctional relationships, it can be concluded that heat-induced proteotoxic stress in human neuronal cell models is accompanied by transcriptional and translational reprogramming, mediated through eIF2 modulation.

### 3.2. Proteostasis Maintenance/Cellular Stress Response

Based on our commonality criteria, this category includes molecular pathways associated with the biological processes of Cellular Response to Stress, Cellular Response to heat stress, Autophagy, and Programmed Cell Death. *Unfolded protein response* is an endoplasmic reticulum (ER) stress-buffering signaling network that maintains protein homeostasis [56]. The unfolded protein response is triggered when the ER protein folding capacity is overwhelmed, for example, by oxidative stress [57,58]. Thus, unfolded protein response activation in heat-induced proteotoxic stress response is not surprising (Figure 12b, block 1), considering the relation between thermal perturbation and protein folding and aggregation. *Chaperone-mediated autophagy (CMA)* is an intracellular lysosomal proteolysis pathway that facilitates the selective degradation of cytosolic proteins. Both *Chaperone- Mediated Autophagy* and *Chaperone-Mediated Autophagy Signaling* Pathways are positively associated with stress response (Figure 12b, blocks 2,3), thus signifying the role of CMA in protein clearance during heat-induced proteotoxic stress. CMA’s role in pathological neuroprotection [59,60] and oxidative stress inducibility [61] are well known; thus, its relevance to heat-induced proteotoxic stress will need further exploration. Related to chaperone-mediated autophagy is *Aggrephagy*, the autophagic dissolution of selective irreparable proteins [62,63]. The activated state of Aggrephagy in heat-induced proteotoxic stress models points toward a substantial irreversible protein unfolding and subsequent aggregation, hence the requirement (Figure 12b, block 4). The *BAG2 Signaling Pathway* activation further augments the function of multiple protein aggregate clearing mechanisms in these neuronal proteotoxic models. Bcl2-associated athanogene 2 (BAG2) is a crucial part of the distinct phase-separated membrane-less organelle, distinct from stress granules and other condensates, and promotes a ubiquitin-independent client degradation [64]. In addition to a direct role in aggregate dissolution, BAG2 functions as a neuroprotective upstream regulator of the PINK1/PARKIN signaling pathway in an in vitro Parkinson’s disease model [65]. BAG2 Signaling Pathway activation (Figure 12b, block 5) in mild and extreme conditions points towards a BAG2-mediated role in heat-induced proteotoxic stress. Two Reactome pathways, *Cellular response to heat stress* (Figure 12b, block 6) and *Cellular response to hypoxia* (Figure 12b, block 7), are also actively functionally associated with the stress response. The proteins compromising these pathways are chaperones and associated proteins, thus highlighting a requirement for proteostasis imbalance ameliorating mechanisms. Based on the activation of these pathways, heat exposure is likely accompanied by the induction of considerable proteotoxic stress in neurons, which requires multiple clearance mechanisms.

### 3.3. Intra- and Intercellular Transport

*Gap junction trafficking and regulation* pathway association signify the role of gap junction intercellular communication and its regulation (Figure 12b, block 8). Gap junctions are intercellular channel-forming structures that facilitate direct communication, play a role in neuronal differentiation and maintenance, and contribute to post-injury functional recovery [66,67,68]. A cytoprotective role for gap junction intercellular communication in hyperthermia in human fibroblasts has been reported [69]. *Kinesins* are a molecular motor protein superfamily that participates in the intracellular anterograde transport of cargo like mRNAs, protein complexes, and membranous organelles [70,71]. The activation of the kinesin pathway (Figure 12b, block 9) demonstrates the significance of this protein family in neuronal heat-induced proteotoxic stress response. While kinesin activation occurs in response to mild and extreme heat, *RAB geranylgeranylation* is an extreme stress response pathway (Supplementary Table S8). Rab GTPases play a role in intracellular membrane trafficking through their diverse role in facilitating membrane-bound organelle transport [72]. Geranylgeranylation is a post-translational covalent modification critical in cell membrane fusion [73]. Thus, intra- and intercellular transportation is vital to heat-induced proteotoxic stress.

### 3.4. Cellular Signaling

Our analysis identifies forty-four associated signaling pathways. Twenty-three of these fit our commonality criteria, meaning they are functionally associated with mild and extreme heat stress. A stress dose-specific activation/inhibition pattern is identifiable in these signaling pathways (Figure 12c,d). A typical example of this behavior is in Rho GTPases and their regulation through *RhoGDI Signaling* (Figure 12d, Block 1). Rho GTPases are regulated through guanine nucleotide exchange factors (GEFs), GTPase activating proteins (GAPs), and GDP dissociation inhibitors (GDIs) [74] Rho GDIs regulate Rho GTPases both positively and negatively [75,76]. Rho GDIs maintain Rho GTPases in an inactive complex, and an increase in free GDI correlates to an overall decrease in Rho activation [75]. From our analysis, RhoGDI is dose-dependent in maintaining Rho GTPase activity. In mild heat stress (Figure 12d, extreme left column), the inhibition of RhoGDI signaling leads to Rho GTPase activation, which is almost the opposite of extreme stress (Figure 10d, extreme right column). Rho GTPases play multiple and significant roles in neuronal physiology and function regulation [77]. Therefore, the tight regulation observed during the stress response is not surprising. *RhoA Signaling* is a conspicuous pathway for its time-dependent behavior during the stress response with high activation at 0H in mild (*z* = 0.302) and extreme (*z* = 1.314) heat stress and inactivation at 6H (MI: *z* = −0.378, EX: *z* = −1.314). RhoA activation is a hallmark of CNS disorders, spinal cord injury (SCI), and neurodegenerative diseases like Parkinson’s disease, Huntington’s disease, Alzheimer’s disease, and amyotrophic lateral sclerosis with neuro-apoptotic and neuroinflammatory roles [78,79]. The high positive activation at 0H in mild and extreme stress makes it a potential stress marker in heat-induced proteotoxic stress. 

Twenty-one pathways are exclusively positively associated with response to extreme heat stress (Figure 12e). In addition to the *RhoGDI Signaling* discussed above, some pertinent signaling pathways include *Signaling by FGFRs*, *Sirtuin Signaling Pathway,* and *Beta-catenin-independent WNT signaling*. Fibroblast growth factors (FGFs) are key signaling molecules in brain development that, in the adult stage, primarily have a homeostatic role in tissue repair and cellular proliferation [80]. Like FGFs, sirtuins are important signaling-modulating proteins with important roles in functions like cellular response to stress, CNS homeostasis, and normal brain function [81,82]. Wnt/β-catenin signaling is critical to neuronal survival and neurogenesis and functions in regulating synaptic plasticity, and its suppression is correlated with Alzheimer’s disease [83,84]. An elaborate and individual discussion around their relevance is beyond the scope here; nonetheless, the diversity of signaling pathways during the heat-induced proteotoxic stress response reported here lays an elaborate platform for a further exploration of their physiological significance and clinical implications. 

Conclusively, this study generates significant information regarding the proteome-level stress response to heat-induced proteotoxic stress in in vitro models of human neurons. Two considerations should be kept in mind. One, the stress response time point EX-0H is offset by an hour compared to MI-0H because of the higher exposure time for extreme heat stress. Thus, the stress response outcomes are not fully time-synchronized. Choosing an additional time point at six hours into stress response helps bridge this gap comprehensively. In IPA Downstream Effects analytic, the activation z-core is not an absolute measurement but a statistical parameter; hence, these results and interpretations require further targeted mechanistic and functional studies. Nonetheless, a significant pathway, functional, and disease association of DEPs in our neuronal models with processes like *p Protein f Folding and Protein Synthesis, Nervous System Development and Function*, and *Neurological Disease*, along with the activation of stress-specific and neuroprotective signaling pathways, establishes a firm relationship between heat-induced proteotoxic stress and neurological disorders while laying the platform for further mechanistic and translational studies.

## 4. Materials and Methods

### 4.1. Cell Culture and Differentiation

Human medulloblastoma Daoy [HTB-186] and human neuroblastoma SH-SY5Y [CRL-2266] were obtained from the American Type Culture Collection (ATCC), Manassas, VA, USA. The maintenance medium included a complete growth medium (DMEM-F12) containing 10% fetal bovine serum (FBS; Life Technologies, Grand Island, NY, USA) and 4 mM L-glutamine, 100 μg/mL streptomycin, and 100 U/mL penicillin. Cells were maintained at 37 °C in 100 mm dishes in a humidified 5% CO_2_ atmosphere. Neuronal differentiation was induced by treating SH-SY5Y neuroblastoma with 10 μM all-trans-retinoic acid (R265; Sigma Aldrich, St. Louis, MO, USA) for at least 72 h under serum-free conditions [23,85].

### 4.2. Heat Stress Induction

Approximately 60% of confluent cells were subjected to heat stress induction by being transferred from 37 °C to the analysis temperature. At zero time, all dishes (with 60–65% confluent cells) were transferred to the heat stress temperature except the control, which continued growing at 37 °C. Mild heat stress involved “exposure temperature” [ET] of 42 °C for an exposure duration of 1 hr. Extreme heat stress involved an [ET] of 44 °C for an exposure duration of 2 h. Cells were harvested at a designated time (0 h and 6 h) post-stress, washed twice with PBS, and trypsinized. Cells were centrifuged at 4000× *g* for 5 min, the supernatant was discarded, and pellets were frozen for subsequent analysis.

### 4.3. Protein Extraction and Concentration Determination

Protein extraction was performed using RIPA Lysis and Extraction Buffer (Thermo Scientific™, Rockford, IL, USA, Cat. No. 89900). Cells were resuspended in RIPA buffer, vortexed, and incubated on ice for 30 min before centrifugation at 10,000× *g* for 20–30 min at 4 °C to separate cell debris. Quantification was performed using Pierce™ BCA Protein Assay Kit (Thermo Scientific™, Rockford, IL, USA). Protein concentrations were determined using a 96-well format and evaluated by referencing a standard such as bovine serum albumin (BSA).

### 4.4. Proteomics

#### 4.4.1. In-Solution Protein Digestion and Peptide Preparation for MS Analysis

As previously described, the whole cell lysate of a complex protein mixture in sample groups was processed for in-solution trypsin enzyme digestion [86,87]. Briefly, solubilized protein from each sample group was adjusted for protein concentration in RapiGest SF buffer (Waters, Manchester, UK). From each sample, 100 μg total protein quantity was heat-denatured at 80 °C for 15 min. Proteins were reduced in 10 mM DTT at 60 °C for 30 min and alkylation in 50 mM Iodoacetamide for 40 min in the dark at room temperature. All samples were digested using sequence-grade trypsin (Promega) at a ratio of 1:50 (*w*/*w*) and left overnight at 37 °C. The enzymatic digestion reaction was halted with 37% hydrochloric acid, and clear supernatant was carefully removed following centrifugation at 13,000 rpm for 10 min at 8 °C. All samples were diluted in 0.1% formic acid and spiked with alcohol dehydrogenase (ADH, P00330) for absolute quantification before mass spectroscopic analysis.

#### 4.4.2. Quantitative Expression Proteomics by Label-Free Liquid Chromatography–Mass Spectrometry

The protein identification of all samples used one-dimensional (1D)-Nano Acquity liquid chromatography coupled with Synapt G2 HD Mass Spectrometry instrument (Waters, Manchester, UK). Pre-analysis instrument calibration and optimization parameters were conducted using the MassLynx tune page, as previously described [86,87]. Briefly, detector setup and mass calibration were conducted using 2 ng/L of leucine enkephalin 500 fmol [Glu] 1-fibrinopeptide. All samples were run in Trizaic Nano Source, in resolution and positive ion mode nanoESI in the AcquityTM HSS T3 85 µm× 100 mm Trizaic Nano tile column (Waters, Manchester, UK). The MS data were acquired in an m/z range of 50–2000 Da with a gradient acquisition run time of 120 min using data-independent acquisition (DIA)/ion mobility separation experiments (HDMSE), (MassLynx version. 4.1, SCN833; Waters, Manchester, UK). 

#### 4.4.3. Data Analysis 

The acquired raw MS data were processed, and database searching was conducted by Progenesis LC-MS proteomics data analysis software (Progenesis QIfP V4.0 (Waters, Manchester/Nonlinear, Newcastle, UK)) as previously described [86,87]. The acquired list of peptide ions was analyzed using the non-redundant UniProt/SwissProt human-specific (Homo sapiens) protein sequence database for achieving protein identification (www.uniprot.org, accessed on 10 September 2023). We applied multivariate statistical analysis to the generated data. Proteins with marked expression change (ANOVA) with a *p*-value < 0.05 and at least ≥2-fold were considered statistically significant. Additionally, we applied measures to overcome the bias of multiple testing leading to a False Discovery Rate (FDR) using an adjusted p-value or q-value calculated based on the algorithm in the licensed Progenesis QIfP program.

### 4.5. Bioinformatics Analysis of DEPs and Molecular Pathway Analysis

The quantitative protein data were imported into IPA software (Ingenuity^®^ Systems, [88,89]) to identify the protein–protein interactions and their functional implications. IPA is a web-based application that enables the interpretation and significance of DEPs by identifying their association with molecular canonical pathways and predicting their biological functions downstream. IPA uses Fisher’s exact test and calculates a *p*-value for each category. A *p*-value < 0.05 indicates statistical significance. IPA also estimates an activation Z-score to infer the likely activation state of the canonical pathway and its biological function. Protein networks were additionally analyzed utilizing STRING [90]. TBtools was used for developing visuals and images [91].

## Figures and Tables

**Table 1:** Thirty-two common DEPs identified through global proteome-wide analysis of heat-induced proteotoxic stress response in Daoy, SH-SY5Y cells, and differentiated SH-SY5Y neuron-like cells.

No	Name	Annotation
1	MAGED2	Melanoma-associated antigen D2
2	NUDT21	Cleavage and polyadenylation specificity factor subunit 5
3	ANP32E	Acidic leucine-rich nuclear phosphoprotein 32 family member E
4	ATXN2L	Ataxin-2-like protein
5	HSPB1	Heat shock protein beta-1
6	RCN2	Reticulocalbin-2
7	RPS15	Ribosomal protein S15
8	ELOC	Elongin-C
9	CMPK1	UMP-CMP kinase
10	GRN	Paragranulin
11	MIF	Macrophage migration inhibitory factor
12	KPNA2	Importin subunit alpha-1
13	EIF4A3	Eukaryotic initiation factor 4A-III
14	MCM4	DNA replication licensing factor MCM4
15	FEN1	Flap endonuclease 1
16	PPM1G	Protein phosphatase
17	NUTF2	Nuclear transport factor 2
18	PUF60	Poly(U)-binding-splicing factor PUF60
19	MYL6B	Myosin light chain 6B
20	KRT3	Keratin
21	ADSS2	Adenylosuccinate synthetase isozyme 2
22	TARDBP	TAR DNA-binding protein 43
23	TPM3	Tropomyosin alpha-3 chain
24	IRGQ	Immunity-related GTPase family Q protein
25	MATR3-2	Matrin-3
26	HSPA1B	Heat shock 70 kDa protein 1A
27	HIBADH	3-hydroxyisobutyrate dehydrogenase
28	GSTO1	Glutathione S-transferase omega-1
29	H4C6	Histone H4
30	TMPO	Lamina-associated polypeptide 2
31	DPY30	Protein dpy-30 homolog
32	DDX3X	ATP-dependent RNA helicase DDX3X

**Table 2:** Cell-wise, condition, and stress response time point-specific proteins. MI: mild heat stress; EX: extreme heat stress.

	MI-0H-6H	EX-0H-6H	MI-0H- EX-0H	MI-6H- EX-6H	MI-0H- EX-0H-6H	MI-6H- EX-0H-6H
**Daoy**	ALDOB, S100A10, SAMD9	OTUD7B, SH3GL2, DPY30, PRPF19, AASDHPPT, INPPL1, PWWP2B, SRSF11, SYT1, CCT2, MAP2K4, UBE2K, CHCHD3, KIRREL1, NCF2, ERH, GPI, RPLP0, LMNB1, LDLR, RBBP7, ANP32B, STRIP2, LTF, SNU13, SFTPD, HMGXB4, RPL9, NDUFA5, UBXN1, SOD1, PNP, P162, GAN, CDH17, ARHGDIB		TMPO, CHORDC1, PDLIM1, ERMARD		THSD7A, ZNF830, SF3A1, NEFL, PKN2
**SH-SY5Y**		ECHS1, SSRP1, KPNA4, GLO1, PFAS, HSPA13, ASL, LSM14B, NAMPT, GNAI2, GSTO1, TUBAL3, BLMH, HSPA6, PEA15, HOXB4, FIS1, CYB5B, ARHGAP1, CCNB2, SURF4, TPPP3, CISD2, COPA, CKB, CLIC5, VDAC3, AKR1A1, DCTN2, MTX2, PRR23A, IPO7, TUBG1, DOHH, USP39, ATP5PB, KRT3, NUDCD1, ATAD3A, DIDO1, CPNE7, MAT2A, GSTP1, RCN2, ETF1, UQCRC2, ARL3, PPP2CA, MAGEA10, NOL3, RPS15	SH3BGRL, PRPSAP2, ARPC5L, SRM, APEH, EIF3F, MPDU1, HAT1	EIF6	GSR, PDCD6, PXN, PSMD8, ECH1, STAMBP, PSMA6, BZW2	
**SH(D)**	PRPF19, KLF1, RPL13A, TET3, VAMP5, HMGN2, BANF1, BPIFA4P RNASEK	TUBA3C/TUBA3D, TRIR, BUB3, MYZAP, HADH, L1TD1, NAXE, MPDU1, MAPRE3, KRT76, ATG101, PLEKHA6, PUF60, KIF3C, GSTO1, TWF2, IWS1, RAB4B, MCM6, KIF5C, ATAD2, XPNPEP1, PITPNB, KIF5A, RCN2, EFCAB5, IPO5		CFAP58		

## Data Availability

The mass spectrometry proteomics data have been deposited to the ProteomeXchange Consortium via the PRIDE [92] partner repository with the dataset identifier PXD051826 and 10.6019/PXD051826.

## Supplementary Materials

The following supporting information can be downloaded at https://www.mdpi.com/article/10.3390/ijms25126787/s1.
