# Supplementary material for: Proteomics Analysis of Proteotoxic Stress Response in In-Vitro Human Neuronal Models
Source: Int J Mol Sci. 2024 Jun 20;25(12):6787. doi: 10.3390/ijms25126787 (PMC11204259; doi:10.3390/ijms25126787)
Supplement: Supplementary file 1 [file ijms-25-06787-s001.zip › SUPP FIGURES-REV.pdf]

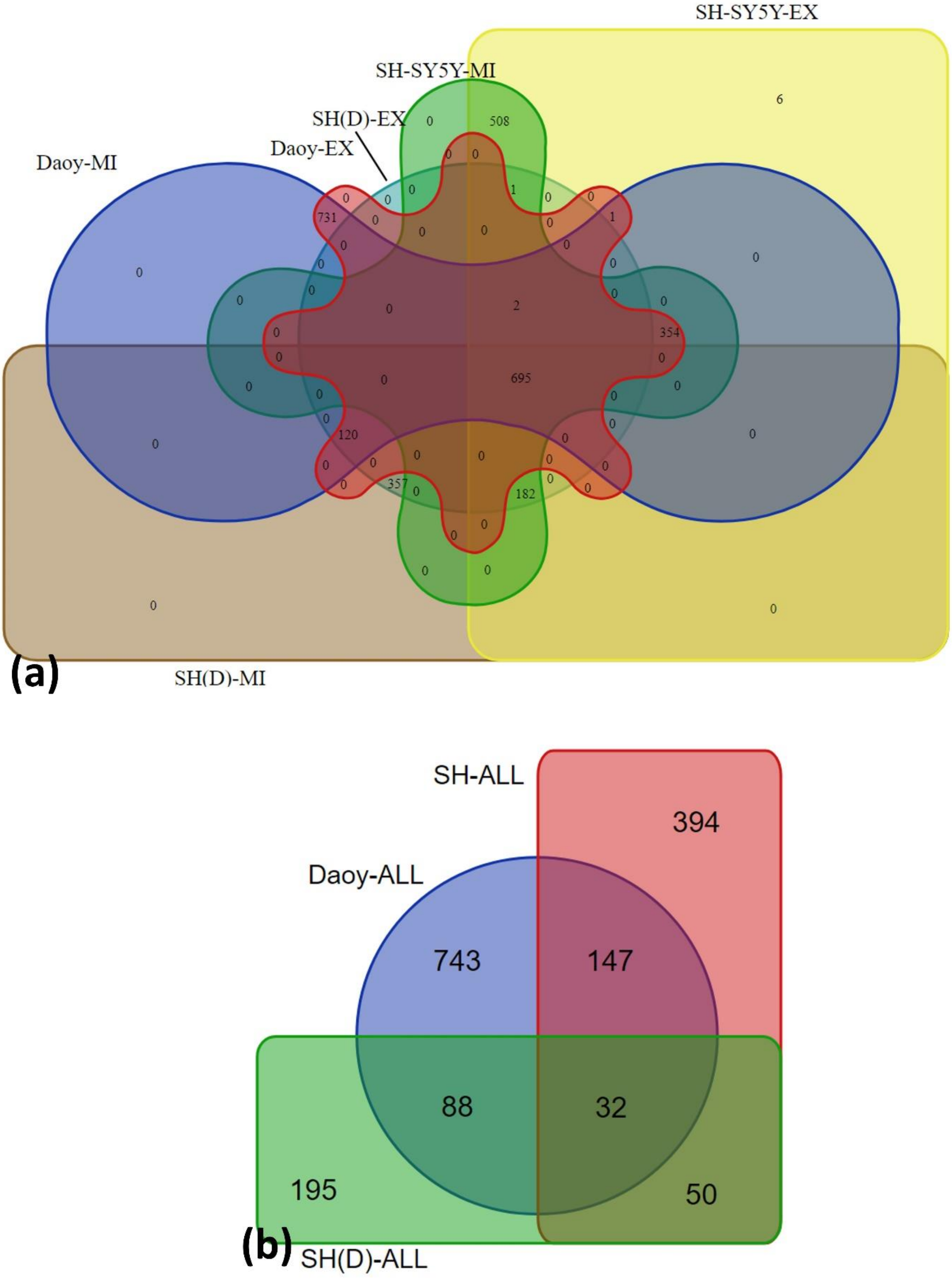

Supplementary Figure S1. Overall proteomics analysis. (a) 695 common proteins are identified among 2957 proteins from the 18 datasets based on three study points in two stress conditions among three cell types. (b) 32 common and significant DEPs (Fold change ≥ 2.0 and p-value ≤ 0.05) among 1010 in Daoy cells, 623 in SH-SY5Y cells, and 365 in differentiated SH-SY5Y cells.

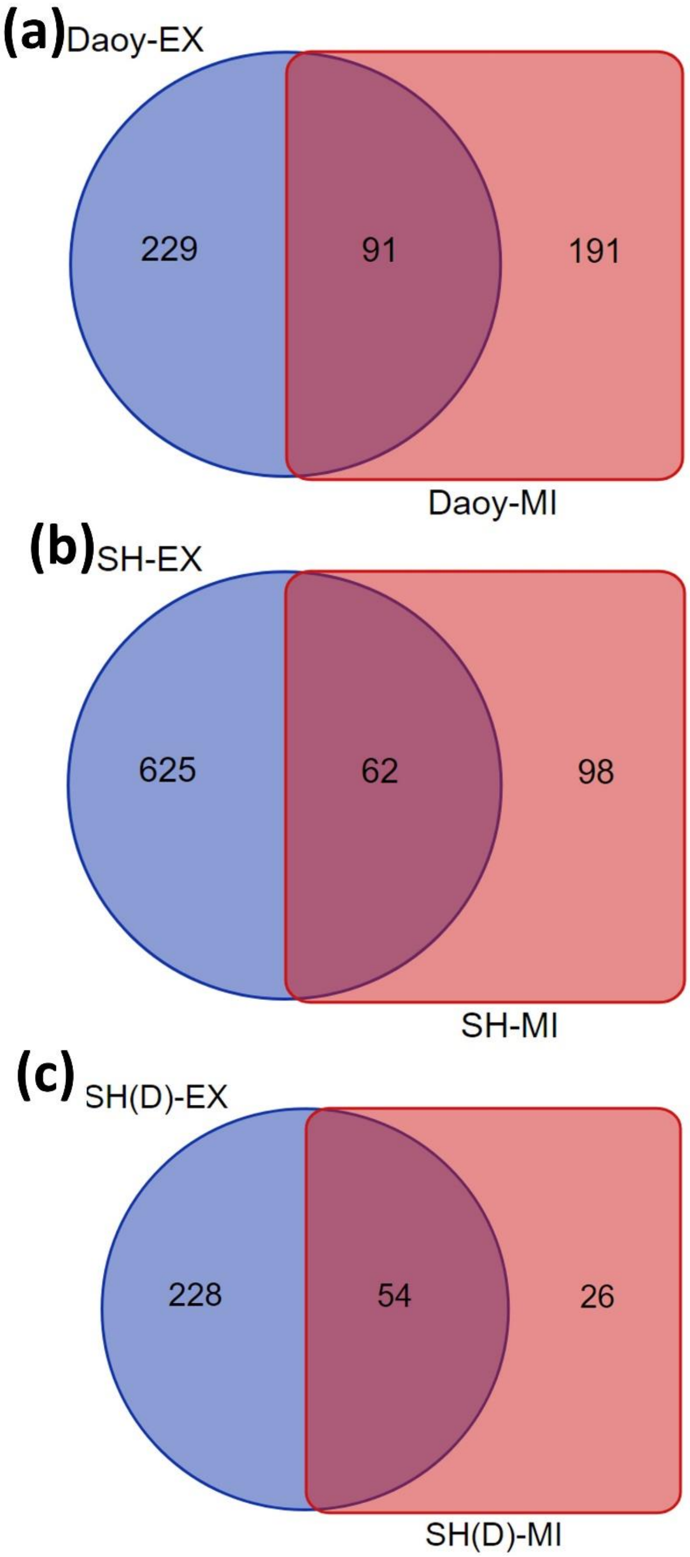

Supplementary Figure S2. Venn Diagrams are used to identify the intra-cellular stress condition-specific proteins. The common proteins are shown in intersections. (d) Ninety-one proteins are common in Daoy. (e) 62 in SH-SY5Y. (f) 54 in differentiated SH-SY5Y cells.

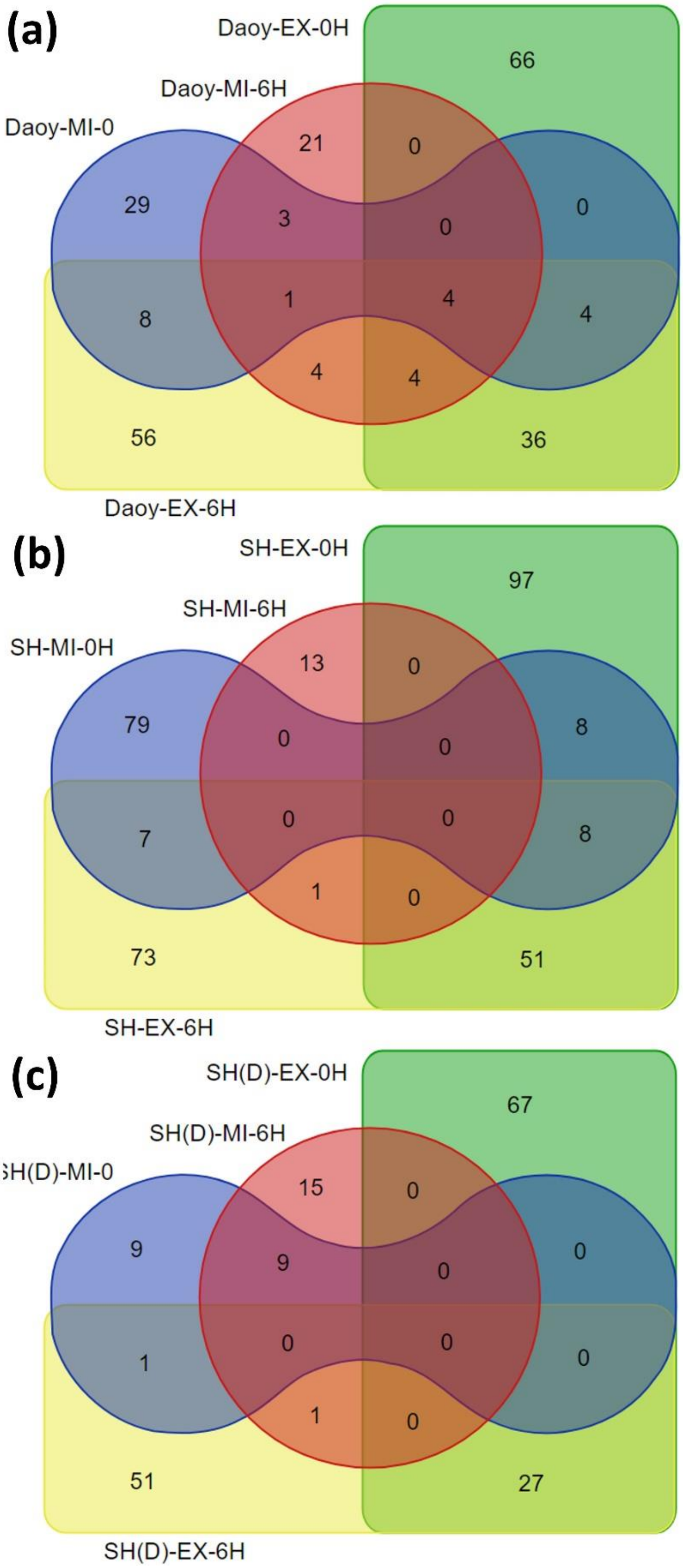

Supplementary Figure S3. Stress condition and stress-response time-point-specific protein specificities. The highest overlap is observed between EX-0H and EX-6H in the intracellular inter-condition comparison. (d) Daoy (36); (e) SH-SY5Y (51); and (f) Differentiated SH-SY5Y cells (27).

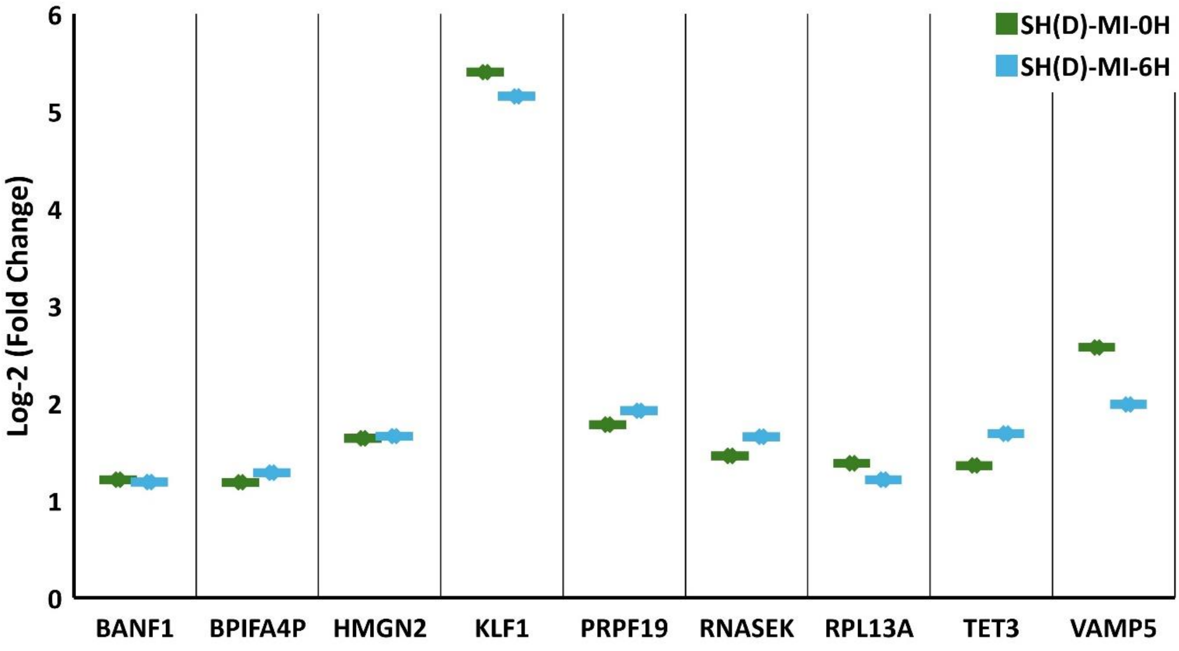

Supplementary Figure S4. Whisker plot displaying 10 common proteins to MI-0H and MI-6H in differentiated SH-SY5Y cells.

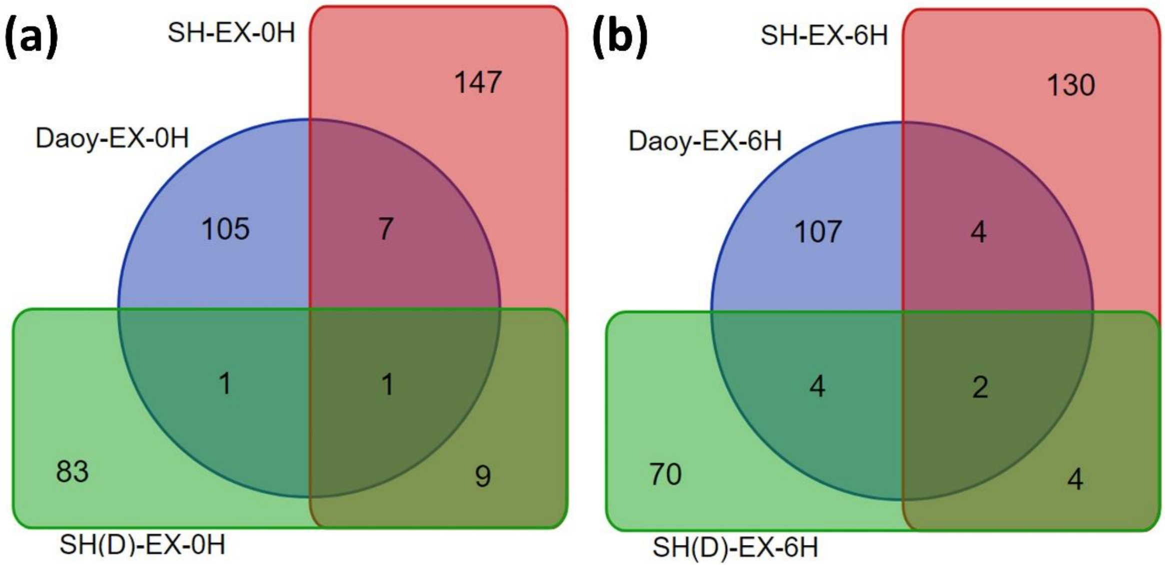

Supplementary Figure S5. Stress condition and stress-response time-point-specific protein specificities. (a) Intercellular EX-0H comparison. One protein is common to the three cell types, and 9 to SH-SY5Y and differentiated SH-SY5Y cells. (b) Intercellular EX-6H comparison. 2 proteins are common to the three cell types, and 4 to SH-SY5Y and differentiated SH-SY5Y cells.

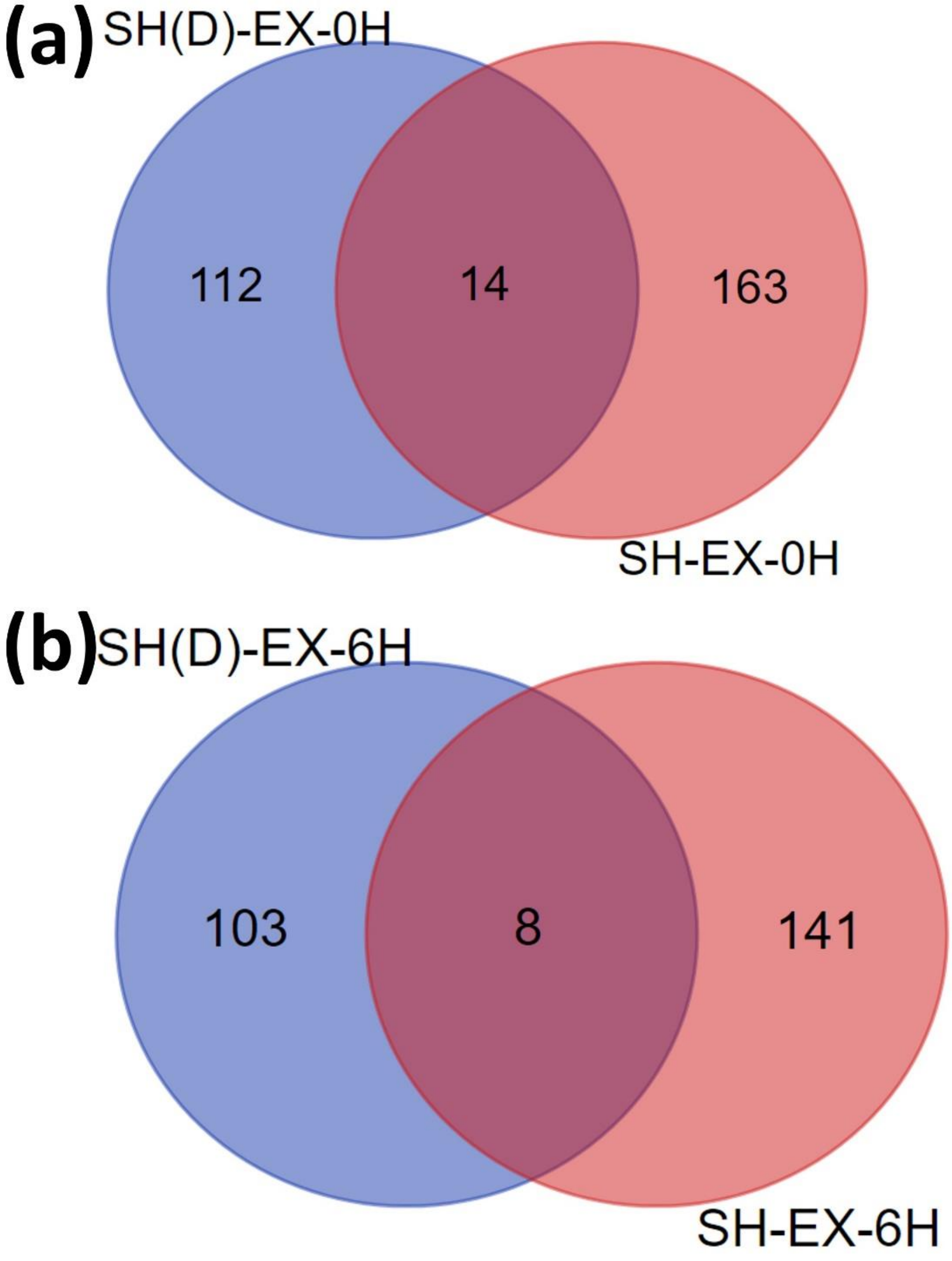

Supplementary Figure S6. Extreme stress condition comparison between SH-SY5Y cells and differentiated SH-SY5Y cells. (a) 0H comparison. 14 common proteins are identified. (b) 6H comparison. 8 common proteins are identified.

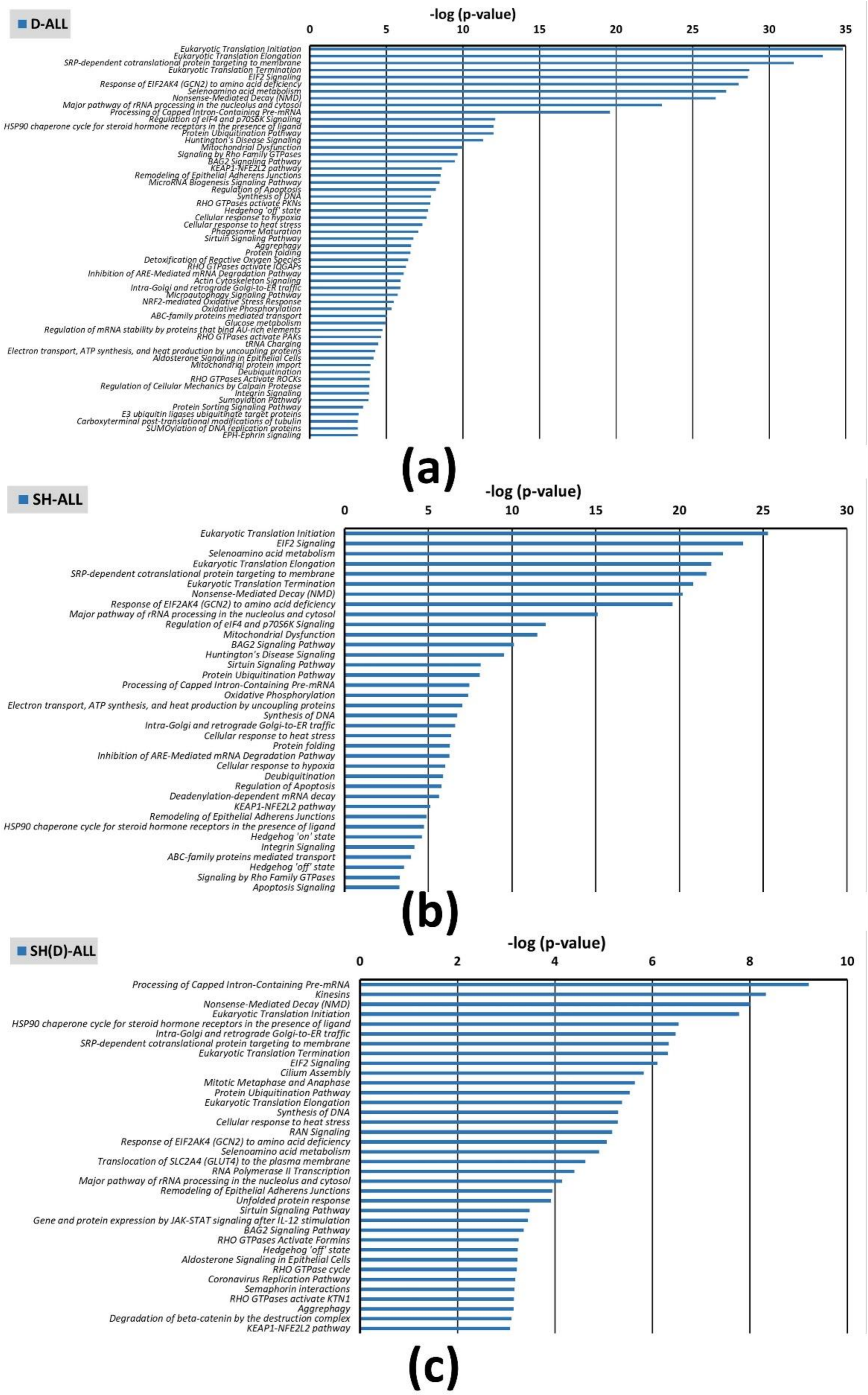

Supplementary Figure S7. Canonical pathways identified through IPA analysis. (a) 56 pathways in Daoy cells were identified at p-value ≤ 0.0001 or a -Log(p-value) of 4. (b) 38 pathways in SH-SY5Y cells were identified at p-value ≤ 0.0001 or a -Log(p-value) of 4. (c) 36 pathways identified in differentiated SH-SY5Y cells were identified at p-value ≤ 0.0001 or a -Log(p-value) of 4.

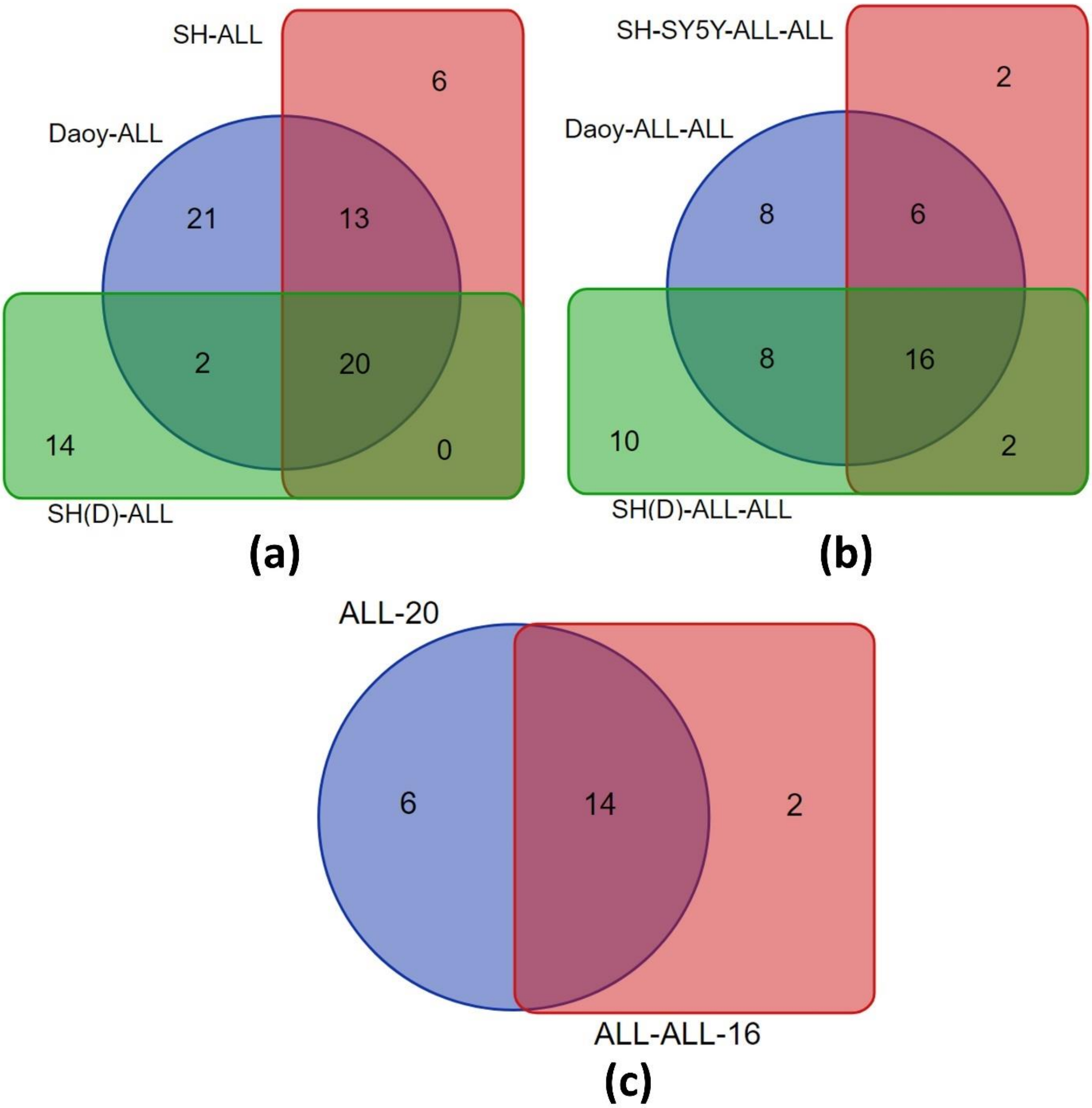

Supplementary Figure S8. Venn diagram-based approach for identifying the common intercellular pathways. (a) The intercellular comparison between the overall stress response pathways (pathway analysis of ANOVA results) identified 20 common pathways to the three cell types. (b) A similar intercellular comparison between the time-dependent stress response pathways identifies 16 common pathways. (c) Common pathways between these two analyses identified 14 stress-response pathways.

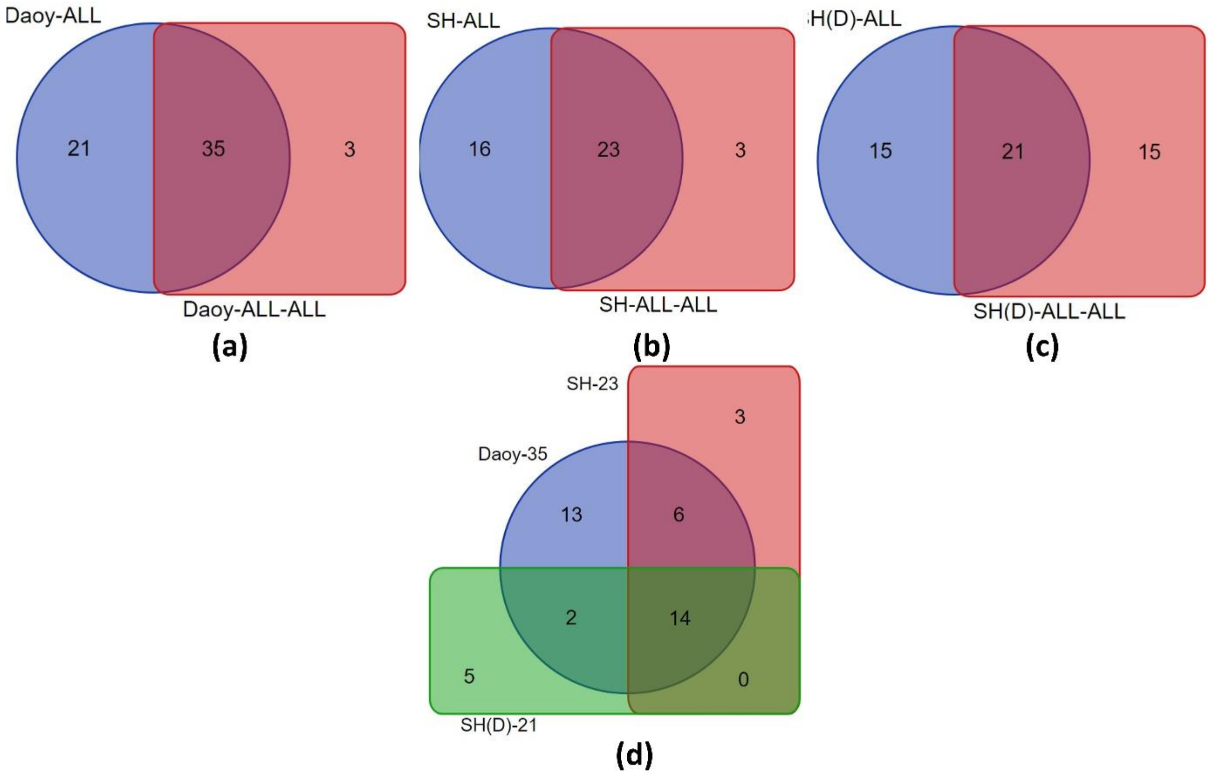

Supplementary Figure S9. Intracellular comparison between pathways identified based on a holistic analysis (ANOVA) of the stress response and pathways associated with a time-dependent response. 35, 23, and 21 common pathways are identified in (a) Daoy, (b) SH-SY5Y, and (c) differentiated SH-SY5Y cells. (d) 14 common pathways were identified from the intercellular comparison of 35, 23, and 21 common pathways identified through (a), (b), and (c).

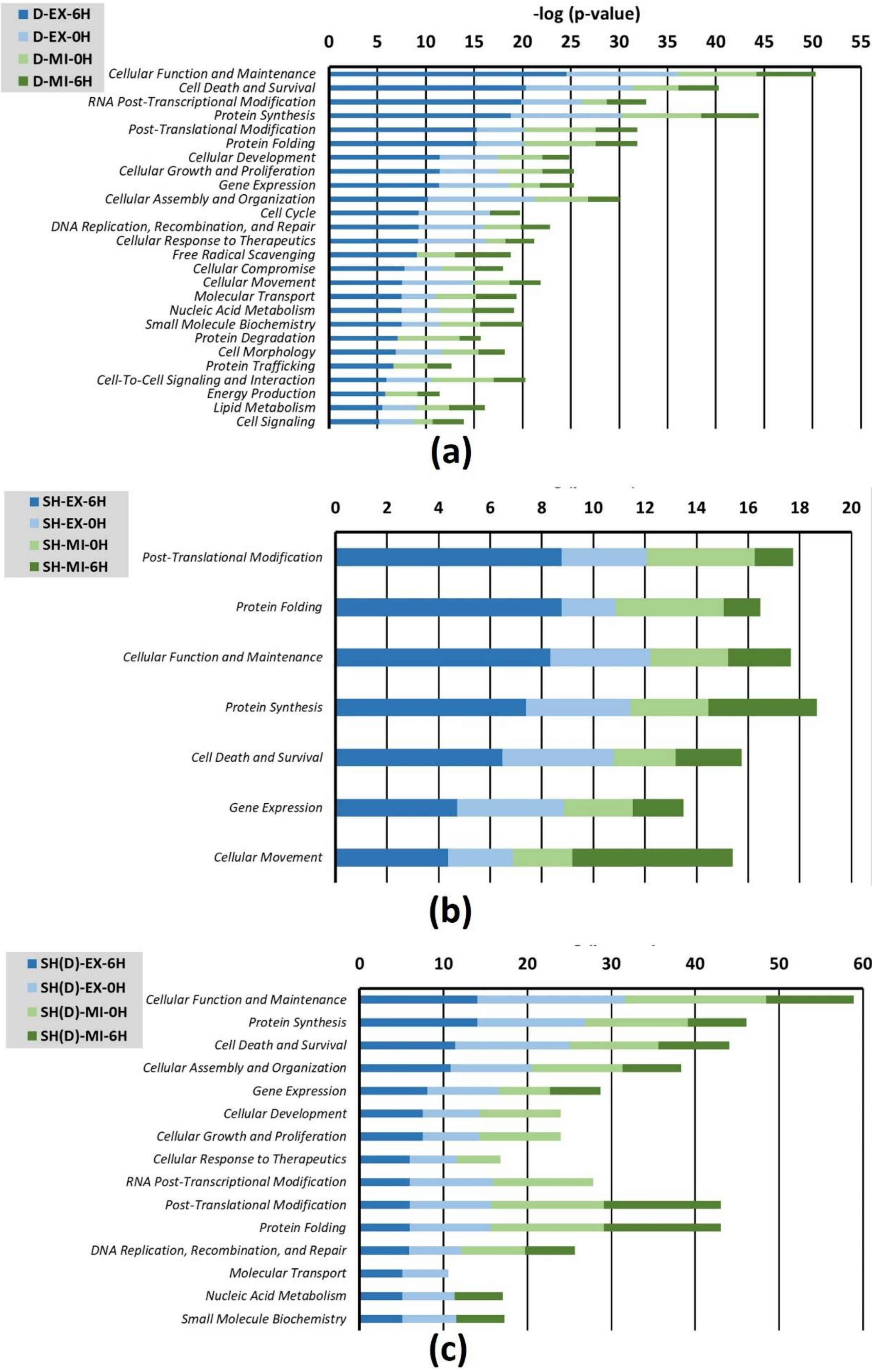

Supplementary Figure S10. Molecular and Cellular Functions analysis in mild and extreme models. 26, 7, and 15 molecular functions are respectively associated at the cut-off association p-value of $5*10^{-6}$ or a -Log(p-value) of 5.3 in (a) Daoy, (b) SH-SY5Y, and (c) Differentiated SH-SY5Y cells at the cut-off association p-value of $5*10^{-6}$ or a -Log(p-value) of 5.3.

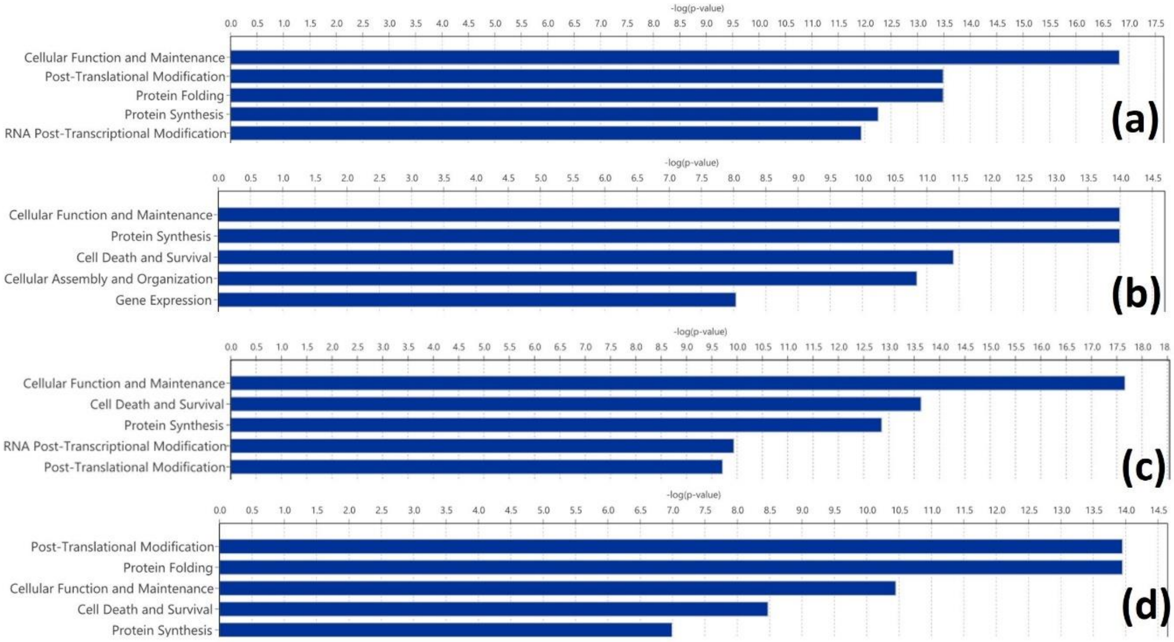

Supplementary Figure S11. Molecular and Cellular Functions associated with the DEPs in differentiated SH-SY5Y cells. The top five functions are plotted for (a) MI-0H, (b) MI-6H, (c) EX-0H, and (d) EX-6H.

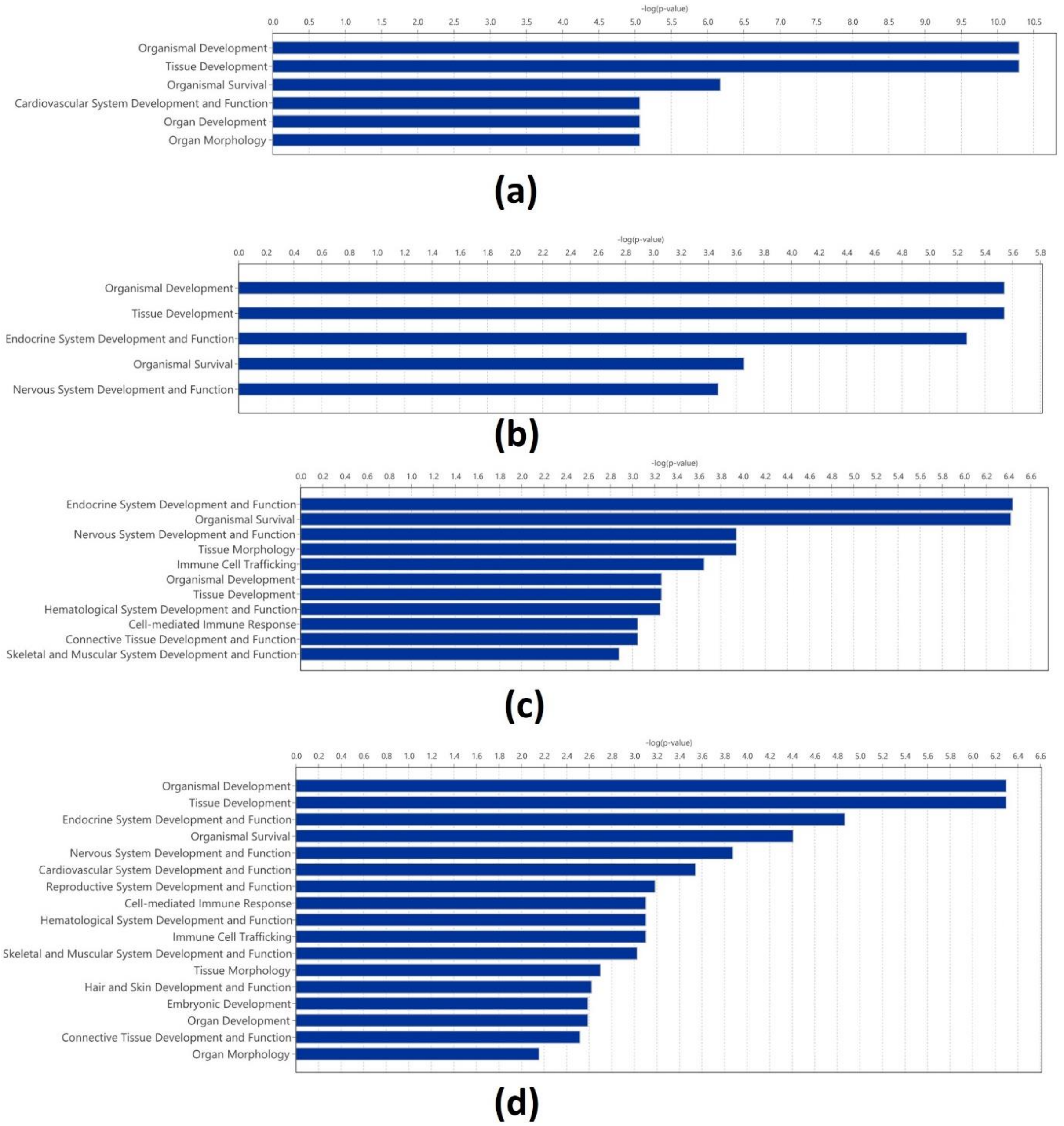

Supplementary Figure S12. Physiological System Development and Function analysis in differentiated SH-SY5Y cells. 6, 5, 11, and 17 pathways of the Physiological System Development and Function are significantly associated with (a) MI-0H, (b) MI-6H, (c) EX-0H, and (d) EX-6H.

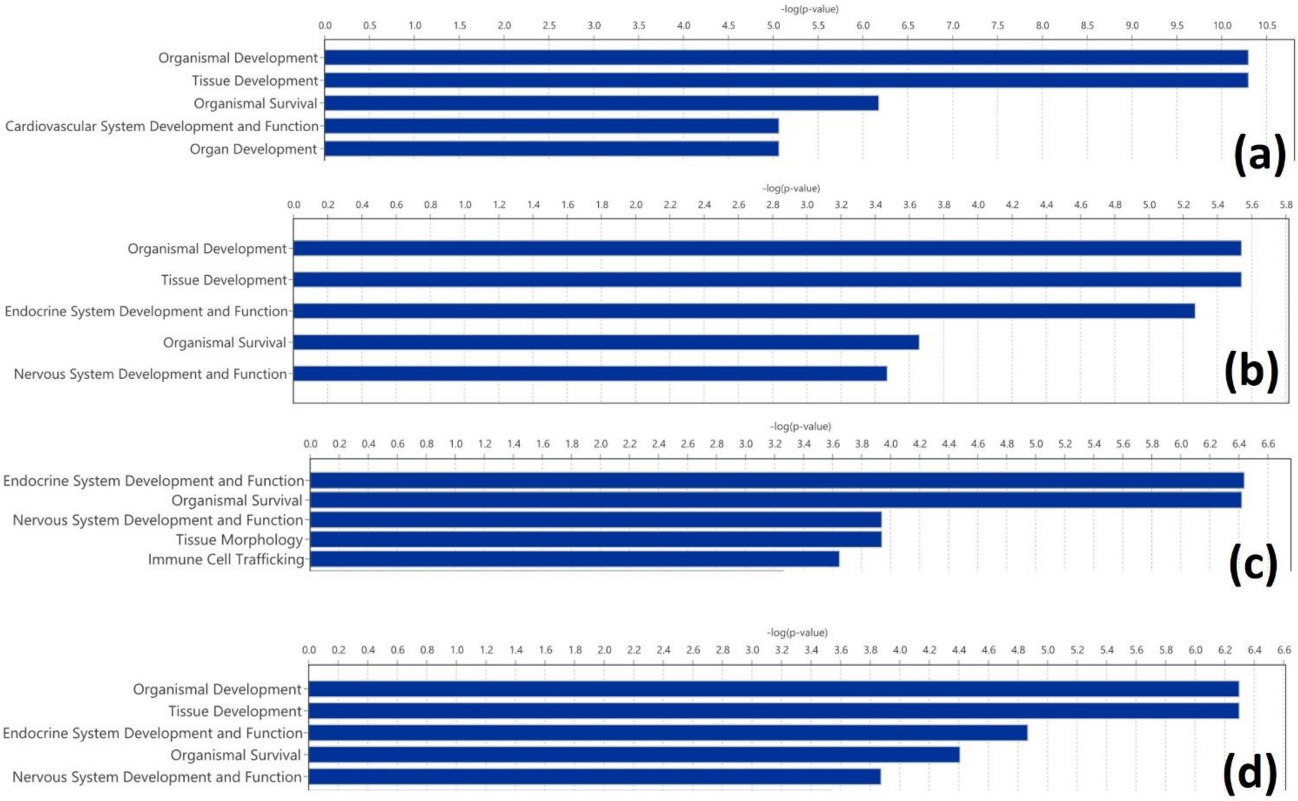

Supplementary Figure S13. The top five Physiological System Development and Function associations with the DEPs in differentiated SH-SY5Y cells: (a) MI-0H, (b) MI-6H, (c) EX-0H, and (d) EX-6H. *Organismal Survival* is a single common pathway associated with the four conditions.

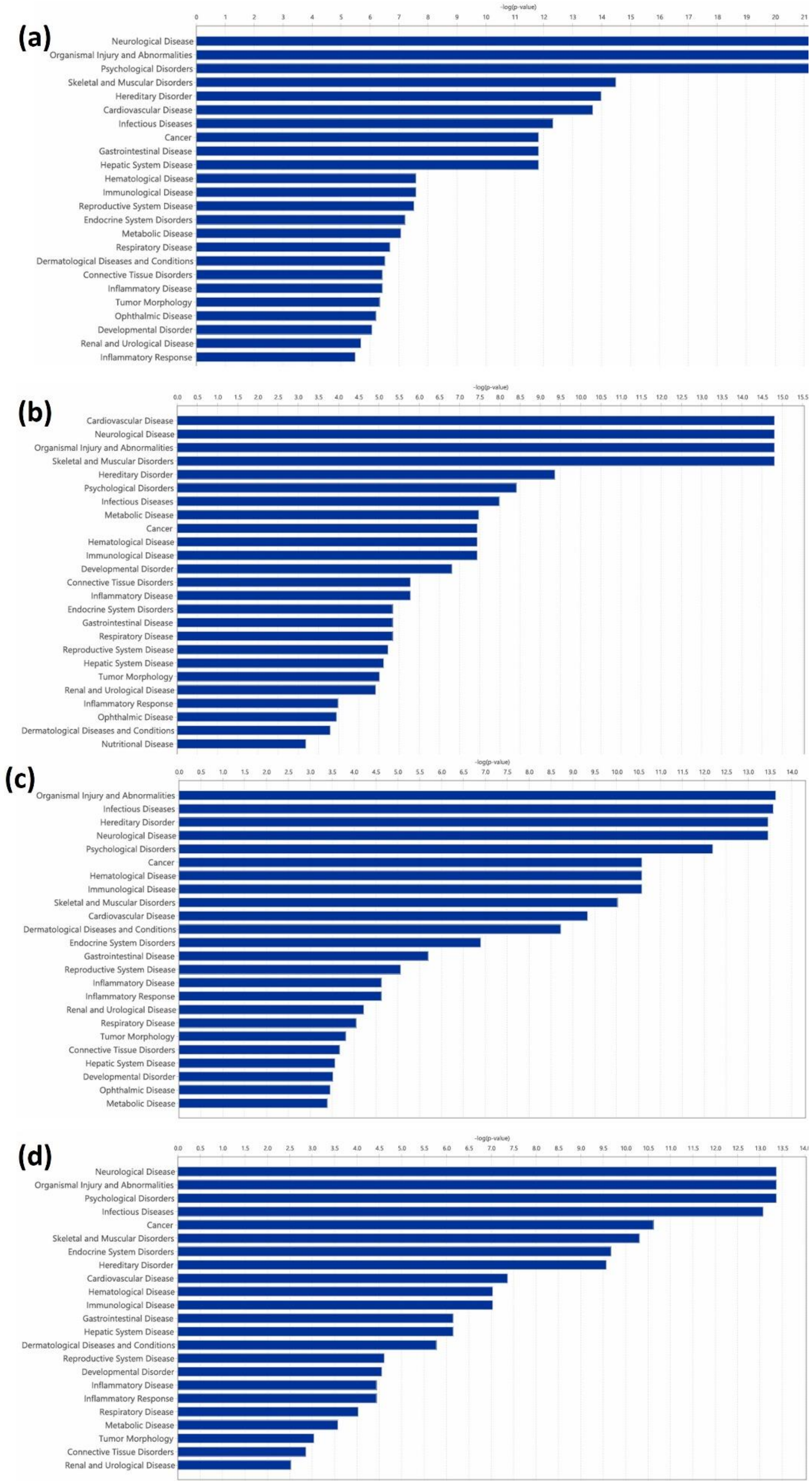

Supplementary Figure S14. Diseases and Disorders analysis in differentiated SH-SY5Y cells. 24, 25, 24, and 25 Diseases and Disorders are significantly associated with (a) MI-0H, (b) MI-6H, (c) EX-0H, and (d) EX-6H.

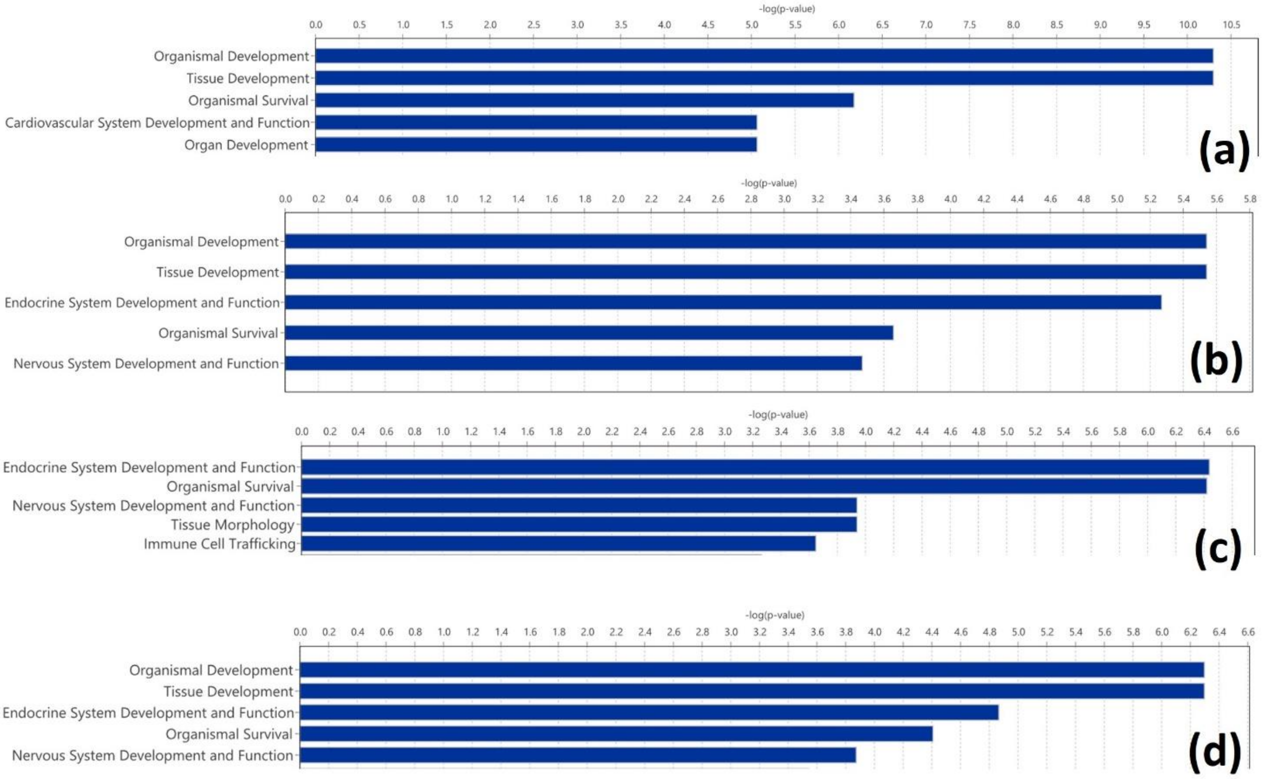

Supplementary Figure S15. The top five Disease and Disorder associations with the DEPs in differentiated SH-SY5Y cells are (a) MI-0H, (b) MI-6H, (c) EX-0H, and (d) EX-6H. Two disorders, *Neurological Disease* and *Organismal Injury and Abnormalities*, are associated with all four analyzed conditions.

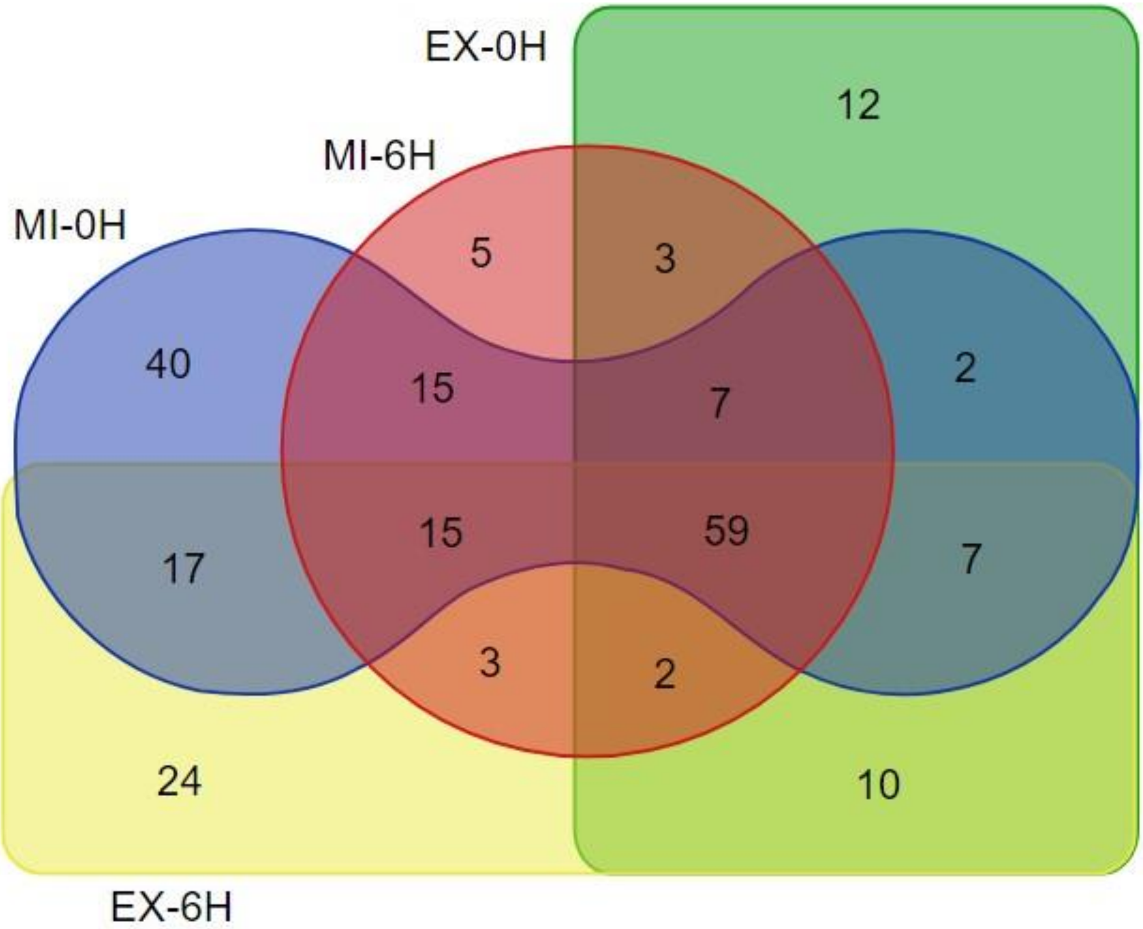

Supplementary Figure S16. Temporal analysis of the significantly associated pathways in differentiated SH-SY5Y cells. Pathways with an association p-value≤ 0.005 or a -Log(p-value) of 2.3 were part of the analysis. Fifty-nine pathways are common to all four conditions.
