# Supplementary material for: Proteomics Analysis of Proteotoxic Stress Response in In-Vitro Human Neuronal Models
Source: Int J Mol Sci. 2024 Jun 20;25(12):6787. doi: 10.3390/ijms25126787 (PMC11204259; doi:10.3390/ijms25126787)
Supplement: Supplementary file 1 [file ijms-25-06787-s001.zip › Supplementary Table S4. DSH-EX0H6H-C27.pdf]

Supplementary Table S4: Upregulated common proteins between 0H and 6H in extreme heat in differentiated SH-SY5Y cells.

			SH(D)-EX-0H		SH(D)-EX-6H	
	Name	Annotation	Log-2 (FC)	-LOG10 (p-value)	Log-2 (FC)	-LOG10 (p-value)
1	ATAD2	ATPase family AAA domain-containing protein 2	3.16	1.77	1.33	1.42
2	ATG101	Autophagy-related protein 101	4.68	2.17	2.63	1.81
3	BUB3	Mitotic checkpoint protein BUB3	3.3	1.61	2.88	1.50
4	EFCAB5	EF-hand calcium-binding domain-containing protein 5	1.37	1.59	1.45	2.04
5	GSTO1	Glutathione S-transferase omega-1	3.69	1.71	2.25	2.16
6	HADH	Hydroxyacyl-coenzyme A dehydrogenase, mitochondrial	2.54	2.39	1.21	1.37
7	IPO5	Importin-5	2.32	1.68	1.05	1.34
8	IWS1	Protein IWS1 homolog	12.82	2.20	6.31	1.32
9	KIF3C	Kinesin-like protein KIF3C	3.14	1.50	1.20	1.55
10	KIF5A	Kinesin heavy chain isoform 5A	3.48	3.33	1.21	3.28
11	KIF5C	Kinesin heavy chain isoform 5C	2.75	2.61	2.78	4.40
12	KRT76	Keratin, type II cytoskeletal 2 oral	4.86	2.26	1.95	2.13
13	L1TD1	LINE1 type transposase domain containing 1	1.59	1.59	4.81	2.74
14	MAPRE3	Microtubule-associated protein RP/EB family member 3	2.41	1.91	1.72	1.67
15	MCM6	DNA replication licensing factor MCM6	1.1	1.48	1.03	1.93
16	MPDU1	Mannose-P-dolichol utilization defect 1 protein	2.8	2.35	1.62	1.68
17	MYZAP	Myocardial zonula adherens protein	4.13	2.53	2.23	1.52
18	NAXE	NAD(P)H-hydrate epimerase	3.77	3.00	2.99	2.53
19	PITPNB	Phosphatidylinositol transfer protein beta isoform	6.86	2.51	3.77	2.40
20	PLEKHA6	Pleckstrin homology domain containing A6	1.35	1.60	2.55	1.30
21	PUF60	Poly(U)-binding-splicing factor PUF60	1.67	2.51	1.40	1.88
22	RAB4B	Ras-related protein Rab-4B	5.21	2.59	3.86	2.05
23	RCN2	Reticulocalbin-2	1.88	1.39	2.29	1.72
24	TRIR	Telomerase RNA component interacting Rnase	1.29	1.84	1.24	1.38
25	TUBA3C	Tubulin alpha 3c	1.55	2.17	1.03	1.80
26	TWF2	Twinfilin-2	1.99	1.74	1.91	3.03
27	XPNPEP1	Xaa-Pro aminopeptidase	3.91	1.85	2.04	2.17
