# Supplementary material for: Proteomics Analysis of Proteotoxic Stress Response in In-Vitro Human Neuronal Models
Source: Int J Mol Sci. 2024 Jun 20;25(12):6787. doi: 10.3390/ijms25126787 (PMC11204259; doi:10.3390/ijms25126787)
Supplement: Supplementary file 1 [file ijms-25-06787-s001.zip › Supplementary Table S6.DSH-EX-0H-U67.pdf]

No	Name	Annotation
1	SCG3	Secretogranin-3
2	IFT74	Intraflagellar transport protein 74 homolog
3	MCF2L	Guanine nucleotide exchange factor DBS
4	ACTR3	Actin-related protein 3
5	SRP68	Signal recognition particle subunit SRP68
6	SPECC1	Cytospin-B
7	DDT	D-dopachrome decarboxylase
8	SEPTIN7	Septin-7
9	PPFIA4	Liprin-alpha-4
10	INTS1	Integrator complex subunit 1
11	CSE1L	Exportin-2
12	KRT20	Keratin
13	NUTF2	Nuclear transport factor 2
14	KIF5B	Kinesin-1 heavy chain
15	RACGAP1	Rac GTPase-activating protein 1
16	ARL6IP5	PRA1 family protein 3
17	CAGE1	Cancer-associated gene 1 protein
18	IDH2	Isocitrate dehydrogenase [NADP], mitochondrial
19	RILPL1	RILP-like protein 1
20	KRT16	Keratin, type I cytoskeletal 16
21	RPRD1A	Regulation of nuclear pre-mRNA domain-containing protein 1A
22	DFFA	DNA fragmentation factor subunit alpha
23	PRPF6	Pre-mRNA-processing factor 6
24	GNA12	Guanine nucleotide-binding protein subunit alpha-12
25	DLST	2-oxoglutarate dehydrogenase E2 component (dihydrolipoamide succinyltransferase)
26	CNDP2	Cytosolic non-specific dipeptidase
27	SOCS7	Suppressor of cytokine signaling 7
28	DPYSL2	Dihydropyrimidinase-related protein 2
29	EPRS1	Bifunctional glutamate/proline--tRNA ligase
30	SYNPO2	Synaptopodin-2
31	NPL	N-acetylneuraminate lyase
32	TRIML2	Probable E3 ubiquitin-protein ligase TRIML2
33	TENT5A	Terminal nucleotidyltransferase 5A
34	MAN2B1	Lysosomal alpha-mannosidase A peptide
35	PAFAH1B2	Platelet-activating factor acetylhydrolase IB subunit beta
36	NRCAM	Neuronal cell adhesion molecule
37	RPS16	Ribosomal protein S16
38	RCC1	Regulator of chromosome condensation
39	CUTA	Protein CutA
40	FAM47A	Protein FAM47A
41	KIDINS220	Kinase D-interacting substrate of 220 kDa
42	SART3	Squamous cell carcinoma antigen recognized by T-cells 3
43	LMNB2	Lamin-B2
44	RPL27A	Ribosomal protein L27a

45	DARS1	Aspartate--tRNA ligase, cytoplasmic
46	RPL9	Ribosomal protein L9
47	KPNA3	Importin subunit alpha-4
48	SDHA	Succinate dehydrogenase [ubiquinone] flavoprotein subunit, mitochondrial
49	TUBA8	Tubulin alpha-8 chain
50	RAB5C	Ras-related protein Rab-5C
51	FIS1	Mitochondrial fission 1 protein
52	LANCL1	Glutathione S-transferase LANCL1
53	PEX14	Peroxisomal membrane protein PEX14
54	PREX1	Phosphatidylinositol 3,4,5-trisphosphate-dependent Rac exchanger 1 protein
55	RAB6A	Ras-related protein Rab-6A
56	NUDT5	ADP-sugar pyrophosphatase
57	CALM1	Calmodulin-1
58	ARL3	ADP-ribosylation factor-like protein 3
59	TMED10	Transmembrane emp24 domain-containing protein 10
60	RAB11B	Ras-related protein Rab-11B
61	PPP2CA	Serine/threonine-protein phosphatase 2A catalytic subunit alpha isoform
62	ATP1A1	Sodium/potassium-transporting ATPase subunit alpha-1
63	DSTN	Destrin
64	CADM1	Cell adhesion molecule 1
65	SLC25A5	ADP/ATP translocase 2, N-terminally processed
66	SURF4	Surfeit locus protein 4
67	LRRC27	Leucine-rich repeat-containing protein 27
