# Supplementary material for: Proteomics Analysis of Proteotoxic Stress Response in In-Vitro Human Neuronal Models
Source: Int J Mol Sci. 2024 Jun 20;25(12):6787. doi: 10.3390/ijms25126787 (PMC11204259; doi:10.3390/ijms25126787)
Supplement: Supplementary file 1 [file ijms-25-06787-s001.zip › Supplementary Table S7.DSH-EX-6H-U51.pdf]

Supplementary Table S7: Fifty-one unique upregulated proteins expressed at EX-6H in differentiated SH-SY5Y cells.

No	Name	Annotation
1	POLA1	DNA polymerase alpha catalytic subunit
2	MPRIP	Myosin phosphatase Rho-interacting protein
3	CMPK1	UMP-CMP kinase
4	SSRP1	FACT complex subunit SSRP1
5	HNRNPR	Heterogeneous nuclear ribonucleoprotein R
6	TJP1	Tight junction protein ZO-1
7	ERLIN2	Erlin-2
8	CHCHD3	MICOS complex subunit MIC19;
9	RAB14	Ras-related protein Rab-14
10	RASGRF2	Ras-specific guanine nucleotide-releasing factor 2
11	SPECC1L	Cytospin-A
12	LMNB1	Lamin-B1
13	H2AX	Histone H2AX
14	DNAJB1	DnaJ homolog subfamily B member 1
15	TUBG1	Tubulin gamma-1 chain
16	PGM1	Phosphoglucomutase-1
17	ADK	Adenosine kinase
18	RBM8A	RNA-binding protein 8A
19	TCEAL3	Transcription elongation factor A protein-like 3
20	RPL27	60S ribosomal protein L27
21	PRAF2	PRA1 family protein 2
22	HSPA6	Heat shock 70 kDa protein 6
23	TMOD3	Tropomodulin-3
24	HSPA4	Heat shock protein family A member 4
25	OTULIN	Ubiquitin thioesterase otulin
26	NMT1	Glycylpeptide N-tetradecanoyltransferase 1
27	TRIM5	Tripartite motif-containing protein 5
28	ENO2	Gamma-enolase
29	CFDP1	Craniofacial development protein
30	HSPA1L	Heat shock 70 kDa protein 1-like
31	PSMD7	26S proteasome non-ATPase regulatory subunit 7
32	TUT4	Terminal uridylyltransferase 4
33	STOML2	Stomatin-like protein 2, mitochondrial
34	SASS6	Spindle assembly abnormal protein 6 homolog
35	MIDEAS	Mitotic deacetylase-associated SANT domain protein
36	HSPA1A	Heat shock protein family A member 1A
37	FHL1	Four and a half LIM domains protein 1
38	ARHGEF40	Rho guanine nucleotide exchange factor 40
39	CRMP1	Dihydropyrimidinase-related protein 1
40	PVR	Poliovirus receptor
41	EIF4A3	Eukaryotic initiation factor 4A-III
42	LGALS1	Galectin-1
43	PGLS	6-phosphogluconolactonase

44	FRS2	Fibroblast growth factor receptor substrate 2
45	SYNJ2BP	Synaptojanin-2-binding protein
46	HSPH1	Heat shock protein 105 kDa
47	RLF	Zinc finger protein Rlf
48	ARHGAP12	Rho GTPase-activating protein 12
49	SAMD9	Sterile alpha motif domain-containing protein 9
50	ASAP3	Arf-GAP with SH3 domain, ANK repeat and PH domain-containing protein 3
51	FANCM	Fanconi anemia group M protein
